# Is happiness for all? The happiness halo effect on coworkers’ perceptions

**DOI:** 10.3389/fpsyg.2025.1653843

**Published:** 2026-01-06

**Authors:** Avital Amir, Zeev Shtudiner, Tal Shavit

**Affiliations:** Department of Economics and Business Administration, Ariel University, Ariel, Israel

**Keywords:** happiness halo effect, employee perceptions, job performance, peerevaluation, employee wellbeing

## Abstract

**Introduction:**

The halo effect is a cognitive bias, in which a specific attribute of an individual influences their overall evaluation. The “halo effect of happiness” refers to a situation in which happier individuals receive a more positive global evaluation. While previous research assessed perceptions of happy employees by supervisors, this study examines how happy colleagues are perceived by their peers. We hypothesize that employees’ happiness would positively predict their perceptions of happy colleagues’ performance, and that affective attitudes and trust would mediate this relationship.

**Methods:**

A sample of 863 employees in the United States completed an online survey assessing their perceptions of happy colleagues’ job performance, affective attitudes and trust, and various measures of happiness.

**Results:**

The findings indicate that there is a happiness halo effect in perceptions of happy colleagues’ performance and reveal that employees’ own happiness predicts these perceptions through their affective attitudes and trust in happy colleagues.

**Discussion:**

Overall, this study highlights that while their colleagues generally perceive happy employees positively, unhappy colleagues may perceive them less favorably. Organizations should consider the diverse needs of all employees to enhance overall wellbeing.

## Introduction

The *halo effect*, first introduced by Thorndike (1920), is a cognitive bias for impression formation, in which the global evaluation of an individual is influenced by specific attributes (Nisbett and Wilson, 1977). In the literature, the halo effect is defined as a “fundamental inability to resist the affective influence of global evaluation on evaluation of specific attributes” (Nisbett and Wilson, 1977, p. 255). It also reflects the incapacity to evaluate each attribute in isolation (Nicolau et al., 2020). According to Kahneman (2011), the halo effect manifests as a predisposition to like or dislike a person as a whole, encompassing attributes that have not been observed. This inclination results in consistent, coordinated judgments, facilitating clearer, easier thought and emotion. Consequently, attributes influencing global evaluation tend to be dominant, central, and easily accessible (Nufer, 2019). Evidence for the halo effect has been identified for a range of attributes (Lemay et al., 2010; Schmidt et al., 2023), including those relevant to employment decisions (Maske et al., 2021; Shtudiner, 2019; Thomas and Reimann, 2023). Given its role in shaping interpersonal evaluations, it is important to explore the halo effect in coworkers’ perceptions. The way coworkers perceive one another can affect employees’ satisfaction and performance (Babin and Boles, 1996; Borgogni et al., 2010), and their interpersonal relations are an integral part of the organizational climate (Su et al., 2024). Understanding these perceptions—and the role of the halo effect in shaping them—highlights a meaningful mechanism that can influence workplace relationships and organizational functioning. This study examines the halo effect in the context of happiness.

Happiness is a notable attribute which contemporary society regards as the ultimate goal and motivation for human action (Diener, 1984; Jain et al., 2019). The significance of happiness is increasingly recognized, causing initiatives to promote it in societies and organizations (Durand, 2018; Tunsi and Bhalla, 2023). Numerous studies have examined how the halo effect influences how happy individuals are perceived, highlighting its impact on attitudes and social perception in economic and organizational contexts (Soderlund and Berg, 2020; Walsh et al., 2018). While previous studies concentrated mainly on the happiness halo effect on employers’ perceptions of employees (Cropanzano and Wright, 1999; Walsh et al., 2023), often emphasizing positive affectivity rather than happiness *per se* (Hosie et al., 2012; Staw et al., 1994), this study explores the happiness halo effect on colleagues’ perceptions of happy employees. Drawing on the concept of relative happiness, which is shaped by social comparison (Chae, 2018; Veenhoven, 1991), we examine the relationship between employees’ perceptions of their happy colleagues’ performance and their own happiness. Specifically, we aim to assess how happy employees are perceived by their colleagues and whether these perceptions relate to their colleagues’ own happiness.

Hence, this study extends the literature on the employees’ happiness halo effect by focusing on colleagues’ perceptions and linking them to colleagues’ own happiness. Given the growing emphasis organizations place on enhancing employee happiness (Tunsi and Bhalla, 2023), understanding how happy employees are perceived by their colleagues is crucial. The study’s findings can inform the development of inclusive policies that address diverse employee needs and foster a positive work environment and overall wellbeing.

The study employed a quantitative approach. A total of 863 employees in the US completed questionnaires assessing their perceptions of happy colleagues’ performance, their affective attitudes and trust toward these colleagues, their own happiness levels, and the importance they place on happiness. Additionally, participants were presented with hypothetical scenarios involving employment decisions. The findings indicate a happiness halo effect for perceptions of happy colleagues’ performance and reveal that these perceptions are predicted by employees’ own happiness through their affective attitudes and trust in happy colleagues.

This paper is organized as follows: The next section provides the theoretical foundation, reviewing the literature on the happiness halo effect and its potential relationship with individuals’ emotional state, as well as perceptions of happy employees’ job performance, leading to the study’s hypotheses. Section 3 outlines the research methodology, including sample characteristics, measures, and the data analysis procedures. Subsequently, the results are presented and analyzed in light of the hypotheses. The final section discusses the main findings and their theoretical and practical implications.

## Theory and hypotheses

### The happiness halo effect

The predominant definition of “happiness” in contemporary psychology is subjective wellbeing (Diener, 1984). This concept encompasses individuals’ evaluations of their lives in terms of both affective and cognitive components of wellbeing (Adler et al., 2017), including frequent and intense positive affective states, a relative absence of negative affective states, and overall life satisfaction (Phillips et al., 2014). Happiness is a highly desirable trait and a central purpose of human action (Diener, 1984; Jain et al., 2019). Attributes that influence global evaluations are typically dominant (Nufer, 2019); consequently, happiness as a dominant attribute may shape global evaluations and induce the halo effect (Walsh et al., 2018). This phenomenon has indeed been identified in numerous studies, which show that happiness predicts higher ratings on unrelated attributes (Belkin and Rothman, 2017; Hong et al., 2025; Liu et al., 2023; Tracy and Beall, 2011) and elicits similar emotions in interaction partners (Methot et al., 2017). Unlike other attributes, the subjective nature of happiness can complicate assessments of others’ happiness at first impressions (Stavrova and Haarmann, 2020). Therefore, the halo effect of happiness may be more relevant in long-term relationships, including those in workplaces.

While numerous studies highlight the positive evaluation of happy individuals—which can be described as a halo effect—potential negative implications have also been suggested (Clark and Monin, 2014). This concept is rooted in specific cultural traditions, suggesting that happiness could evoke envy in others (Uchida and Kitayama, 2009). Based on Klein (1975) psychoanalytical theory, Hamman (2015) argued that witnessing others’ happiness may trigger painful memories of one’s own lost happiness, thereby prompting envy. Similarly, Clark and Monin (2014) suggested that observing others’ happiness can induce self-comparison, causing individuals to feel inadequate and potentially derogate or undermine the happy individual.

Although we do not have direct evidence for the “dark side” of happiness and its impact on individuals (Clark and Monin, 2014), these ideas might align with theories of happiness and social comparison—specifically, the theory that “happiness is relative” (Easterlin, 1974; Veenhoven, 1991) and social comparison theory (Festinger, 1954). The theory that happiness is relative posits not only that happiness is relative but also that it is influenced by subjective, social comparison to others (Chae, 2018; Veenhoven, 1991), and by standards of how life should be, regardless of actual quality of life (Veenhoven, 1991, 2016). Despite criticism of this theory over the years (Veenhoven, 2016), it seems that happiness is indeed partly—though not entirely—relative and might be influenced by social comparison (Chae, 2018; Veenhoven, 1991).

Another theoretical framework aligned with this concept is Festinger’s social comparison theory (1954), which posits that individuals assess their worth in specific areas through comparisons with others. Smith (2000) expanded this theory by delineating outcomes of upward and downward comparisons. Upward comparison, in which another person’s performance surpasses one’s own, can evoke emotions ranging from admiration to envy and resentment. Yada and Ikegami (2014) further noted that when individuals perceive the attribute as achievable, upward comparisons can foster admiration and a halo effect. However, upward comparisons may also lead to envy toward the outperformer, resulting in a compensation effect in which the outperforming person is negatively evaluated in other attributes. Similarly, scholars have pointed out that envy can motivate degeneration of the outperforming person in unrelated dimensions (Cohen-Charash and Mueller, 2007; Van de Ven, 2017; Wert and Salovey, 2004). To enhance self-concept and avoid distress from social comparison, individuals may prefer to compare themselves with those inferior to them (Wood, 1989). Therefore, unhappy individuals might prefer to compare themselves to less happy people, thus avoiding potential pain and distress. Should the happiness of others indeed provoke a negative effect, it might negate the halo effect (Forgas, 2011).

### Perceptions of job performance of happy employees

Job performance, meaning employees’ actions, is essential if organizations are to achieve their goals (Arshad et al., 2023). Several factors influence job performance, including happiness and wellbeing (Arshad et al., 2023; Cheng K. C. et al., 2025; Cropanzano and Wright, 1999). Accordingly, employees characterized by happiness and positive affectivity tend to be positively evaluated by their supervisors (Cropanzano and Wright, 1999; Hosie et al., 2012; Staw et al., 1994), and are considered more employable (Sherman et al., 2025; Sherman and Barokas, 2019). The happiness halo effect may explain this phenomenon (Walsh et al., 2023; Wright and Staw, 1999).

While previous research has examined supervisors’ perceptions of happy employees, some scholars have shifted their focus to consider the issue from colleagues’ viewpoint. Perceptions of colleagues’ performance and involvement can enhance job satisfaction and performance (Babin and Boles, 1996; Borgogni et al., 2010) and frequently are a factor in employee evaluations (Masanja and Rweyemamu, 2020). In the context of happiness, positive affectivity and mood were found to associate with better relationships and more favorable evaluations from coworkers (Ghasemy and Frömbling, 2023; Iverson et al., 1998; Staw and Barsade, 1993), possibly due to the halo effect (Staw et al., 1994). Diener and Tay (2017) suggested that happy employees have better relationships and their coworkers have greater trust in them and are more loyal to them, though this suggestion has not been directly tested. Therefore, our first hypothesis is:

*H1*: Happier employees are perceived by their colleagues as having better job performance.

While literature generally shows overall positive perceptions of positive, happy employees, some studies suggest that happy employees’ performance may not always be received well by colleagues and managers. Barasch et al. (2016) found that peers had negative perceptions of very happy employees, in hypothetical studies. Kalfaoğlu (2024) discussed managers’ fear of an employees’ “happiness overdose.” Other research proposed that employees’ happiness and positivity may lead to envy among their colleagues. For example, recent findings indicate that when an employee expresses gratitude toward a supervisor, coworkers who perceive their own relationship with the supervisor as less favorable may engage in social comparison, leading to feelings of envy and a desire to undermine the grateful employee (Hong and Thoroughgood, 2025). Similarly, Ganegoda and Bordia (2019) suggested that positiveness can trigger social comparison and envy among colleagues. Sherman and Barokas (2019) proposed a similar idea, suggesting that happy female job applicants triggered envy in female employers. Additionally, happiness can sometimes be perceived as inappropriate or inauthentic, leading to reduced trust and respect (Cheshin, 2020), or as ineffective (Kalfaoğlu, 2024), particularly in confrontational situations (Tamir and Ford, 2012) or negotiations (de Melo et al., 2011; Van Kleef et al., 2004). Building on the existing literature, we propose that employees’ personal happiness predicts both their perceptions of their happy colleagues’ job performance and their attitudes toward them.

*H2*: The happier employees are, the more they perceive their happy colleagues as performing their jobs well.

As noted above, reactions to individuals who excel in specific domains may be shaped by emotions stemming from self-comparison, which leads to envy or admiration. While admiration can create an idealized image, envy may lead to derogation of the envied person in unrelated dimensions (Cohen-Charash and Mueller, 2007; Yada and Ikegami, 2014). Therefore, our third hypothesis suggests that emotional attitudes toward happy colleagues mediate the relationship between employees’ personal happiness and their perceptions of happy colleagues’ performance.

*H3*: Affective attitudes toward happy colleagues mediate the relationship between employees’ happiness and their perceptions of happy colleagues’ performance. Specifically, the happier employees are, the more positive their affective attitudes toward happy colleagues, which in turn predicts their perceptions of these colleagues’ performance.

Mauss et al. (2011) noted that individuals may feel disappointed with their own happiness and experience a decline in happiness when they value it highly and see it as attainable. Conceivably, employees who are unhappy and place great importance on happiness may view the presence of happy colleagues as evidence that happiness is achievable, leading to disappointment and decreased happiness. This negative affect may result in more negative impressions of happy employees (Forgas and Bower, 1987) and diminish the halo effect (Forgas, 2011; Yada and Ikegami, 2014). Hence, our fourth hypothesis posits that the relationship between the value employees place on happiness and their perceptions of happy colleagues’ performance is moderated by their personal happiness.

*H4:* Employees’ own happiness moderates the relationship between valuing happiness and perceptions of happy colleagues’ performance, such that the relationship becomes stronger as their happiness increases.

## Materials and methods

### Sample

We conducted an online survey^1^ using Prolific, a platform known for providing efficient, high-quality data suitable for academic research (Douglas et al., 2023). Participants were recruited through Prolific’s standard sampling method, restricted to U.S. residents who met the pre-screening criteria: being currently employed (either full-time or part-time), aged 18–65, and regularly interacting with coworkers in their workplace. Eligible participants could enroll on a “first come—first-served” basis until the target sample size was reached. Participants received monetary compensation via Prolific. A total of 881 US employees completed the survey, with 18 excluded for failing at least one of the two attention questions.^2^ Thus, the final sample included 863 employees (54.8% female; mean age = 38.62).

### Measures

The survey included the following parts:

#### Colleagues’ job performance

Colleagues’ job performance was evaluated using the Role-Based Performance Scale (RBPS) (Welbourne et al., 1998), a widely used measure of job performance (Zacher, 2014). The RBPS employs a multidimensional approach encompassing five components of work:

1. *Job*—Tasks related to job descriptions (e.g., quantity of work produced, output).
2. *Career*—Acquiring skills to facilitate advancement within the organization (e.g., seeking out career opportunities).
3. *Innovation*—Creativity in the job (e.g., coming up with new ideas).
4. *Team*—Collaboration with coworkers to promote organizational success (e.g., working as part of a team and a group).
5. *Organization*—Taking actions to promote the organization beyond what is requ ired.

The 20 items were presented in random order, with four items for each dimension. The RBPS was adapted to assess perceptions of happy colleagues’ performance rather than self-reported performance. Specifically, each item referred to “happy employees” (“The happier employees are, the less/more they…”). To capture both positive and negative perceptions, the items were designed to include bipolar response format, ranging from 1 (*much less*) to 7 (*much more*), allowing to participants indicate whether they perceive happy employees as performing better or worse on each dimension.

Thus, this measure was designed to examine the potential happiness halo effect, reflected in the tendency to evaluate happy colleagues more positively on work-related traits that are not directly related to happiness. The internal consistency of the scale, as measured by Cronbach’s alpha, was high across all five dimensions: Job (α = 0.83), career (α = 0.71), innovation (α = 0.87), team (α = 0.79), and organization (α = 0.82). A sample item: “The happier employees are, the less/more they satisfy the quantity of work output that is officially required.” The full questionnaire is presented in the online Supplementary section 1.

#### Affective attitudes toward happy employees

We used 27 statements to measure affective attitudes toward happy employees, presented in random order. Similar to the RBPS, the statements were adapted to refer to happy employees and were phrased using a bipolar “less/more” structure to capture both positive and negative attitudes. Responses were recorded on a 7-point Likert-scale from 1 (*much less*) to 7 (*much more*). The full scale is presented in the online Supplementary section 2. Four components of affective attitudes were measured:

1. *Affective trust—*Measured using a 5-item scale developed by Dunn et al. (2012), adapted from McAllister (1995) and Johnson-George and Swap (1982). A sample item: “The happier employees in my workplace are, the less/more I would share with them my most outlandish ideas and hopes.”
2. *Cognitive trust—*Measured using a 6-item scale developed by Dunn et al. (2012), adapted from McAllister (1995) and Johnson-George and Swap (1982). A sample item: “The happier employees in my workplace are, the less/more I would take their advice about work.”
3. *Negative affect*—Measured using a 12-item scale developed by Dunn et al. (2012), measuring emotions of envy—based on adaption of research by Schaubroeck and Lam (2004) and Smith et al. (1999)—anger, contempt, and threat. An example item is: “The happier employees in my workplace are, the less/more I feel relatively inadequate.”
4. *Positive Affect*—Measured using a 4-item scale developed by Wayne and Ferris (1990) to gauge liking for subordinates. A sample item: “The happier employees in my workplace are, the less/more I like them.”

The internal consistency of the four subscales, as measured by Cronbach’s Alpha, was high: affective trust (α = 0.72), cognitive trust (α = 0.70), negative affect (α = 0.86), and positive affect (α = 0.84).

#### Happiness

We assessed three components of happiness:

1. *Global life evaluation*—Participants rated their lives on a scale from 0 (*worst possible life*) to 10 (*best possible life*).^3^
2. *Life meaning and purpose*—This scale evaluates participants’ sense of life meaning, according to the eudaimonic viewpoint which defines happiness in terms of life meaning and purpose (Barokas et al., 2022; Ryan and Deci, 2001). Participants rated their perception that things in their life are worthwhile from 0 (*not worthwhile at all*) to 10 (*very worthwhile*).
3. *Affective dimension*—This evaluates positive (enjoyment, exaltation, and smile or laughter) and negative (concern, sadness, depression, and anger) feelings experienced the day before, rated from 0 (*not at all*) to 10 (*very much*).^4^ The internal consistency of the affective dimension was high, with Cronbach’s α = .84 for positive affectivity and 0.87 for negative affectivity.

In addition, job satisfaction, reflecting aspects of happiness that are related to work (Aryanti et al., 2020), was assessed using a Likert scale from 0 (*not at all*) to 10 (*very much*). Job meaning, another indicator of wellbeing (Charles-Leija et al., 2023), was similarly measured from 0 (*not meaningful at all*) to 10 (*very meaningful*). The full scale is presented in the online Supplementary section 3.

#### Valuing happiness

The value attributed to happiness was measured using two items from Mauss et al. (2011). Participants rated the importance of happiness and the extent to which they believe their happiness reflects their life’s worth on a Likert scale from 1 (*strongly disagree*) to 7 (*strongly agree*). Cronbach’s alpha measured for the scale was 0.67. The items are presented in the online Supplementary section 4.

#### Scenarios

To explore how perceptions of happy colleagues’ performance relate to employment decisions, participants were presented with two hypothetical scenarios based on Benjamin et al. (2012). Each scenario offered two alternatives. The first scenario involved a choice between an organization with a happiness-promoting policy and one without, which offered a 10% higher salary. The second scenario required choosing between an organization with happy employees and one with neutral employees that also offered a 10% higher salary. Participants specified their chosen organization and indicated their level of certainty concerning their decision on a three-point scale. The full scenarios are presented in the online Supplementary section 5.

#### Socio-demographic data and other factors

Participants provided socio-demographic information including gender, age, relationship status, children, education, religiosity, health, and annual income, and satisfaction with financial resources. Additional work-related data were collected, including job tenure, attendance requirements, and current job position were collected (the full socio-demographics questions are presented in the online Supplementary section 6).

### Data analysis

Data were analyzed using SPSS 29.0.1. To test the hypotheses, we performed a series of linear regression analyses, as well as mediation analyses following Preacher and Hayes (2008). We used the PROCESS macro program (v.4.2; Hayes, 2022) [models 4 and 1], including bootstrapping 5,000 resamples and 95% confidence intervals. We also conducted *T*-tests to examine the relationship between employment decisions in the scenarios and perceptions of happy colleagues’ performance.

## Results

### Descriptive statistics

Table 1 presents the means, standard deviations, and relative proportions of the participants’ socio-demographic and objective variables.

**TABLE 1:** Means, standard deviations and percentages of socio-demographics features.

Variable	N	%	M	SD
**Gender**				
Male	390	45.2%		
Female	473	54.8%		
**Job position**				
Managerial role	446	51.7%		
Other	417	48.3%		
**Relationship**				
No	231	26.8%		
Yes	632	73.2%		
**Children**				
No	438	50.8%		
Yes	425	49.2%		
Age			38.62	11.08
Education			5.49	1.45
Job tenure			6.24	5.78
Annual income			4.18	1.93
Health			3.85	0.79

Education level was measured by an eight-point scale that included: (1) Some high school or less; (2) High school graduate; (3) Post-high school training; (4) Some college, no degree; (5) Associate degree; (6) Bachelor’s degree; (7) Master’s degree; (8) Doctoral degree. Income was measured by an 8-point Likert scale. Health was measured by a 5-point Likert scale.

Table 2 displays the means and standard deviations of the study variables, reflecting participants’ perspectives on different aspects of life and their job. As shown in Table 2, perceptions of happy colleagues’ performance (job performance and affective attitudes) were significantly (one-sample *t*-test, *p*-value < 0.01) higher than 4 (neutral point) across different domains of job performance, reflecting overall positive perceptions of them (negative affect was significantly < 4).

**TABLE 2:** Means and standard deviations of study variables.

Variable	M	SD	Cronbach’s α
**Job performance**			
Job	5.98	0.75	0.83
Career	5.44	0.92	0.71
Innovation	5.73	0.87	0.87
Team	5.81	0.78	0.79
Organization	5.93	0.79	0.82
**Affective attitudes**			
Affective trust	4.67	0.91	0.72
Cognitive trust	5.27	0.81	0.70
Negative affect	2.82	0.96	0.86
Positive affect	5.66	0.91	0.84
**Happiness and wellbeing**			
Global life evaluation	5.52	1.76	
Life meaning and purpose	6.93	2.28	
Positive affectivity	5.44	2.24	0.84
Negative affectivity	2.83	2.27	0.87
Valuing happiness	5.12	1.26	0.67
Job satisfaction	6.51	2.41	
Job meaning	6.37	2.89	

**FIGURE 1:**
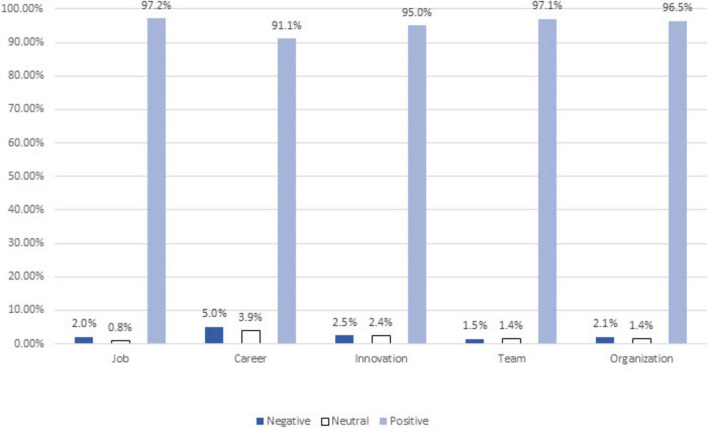
Percentages of negative, neutral, and positive perceptions of happy colleagues’ performance.

Figure 1 presents the percentages of participants expressing positive, neutral, and negative perceptions of their happy colleagues’ performance. As shown in the figure, most participants expressed positive perceptions of happy colleagues’ performance across all performance domains, indicating a happiness halo effect consistent with hypothesis 1.

### Differences in happiness halo effect as predictors of employment decisions

We conducted independent-sample *t*-tests to explore the relationship between employment decisions and the happiness halo effect. Specifically, we examined the differences in perceptions of happy colleagues’ job performance between employees who would choose to work in an organization that promotes happiness or has happy employees versus those opting for an organization that does not promote happiness or where the employees are neither happy nor unhappy but offers a 10% higher salary.

We first analyzed the differences in perceptions of happy colleagues’ performance between participants who chose a happiness-promoting organization and those who opted for a neutral organization (scenario 1). Table 3 presents the *t*-tests results. Altogether, participants selecting the happiness-promoting organization had more positive views of their happy colleagues’ performance in all dimensions than those selecting a neutral organization.

**TABLE 3:** The relationship between employment decisions in scenario 1 and happy colleagues’ job performance dimensions.

Index	Group	N	M(SD)	*T*	*P*-value
Job	Neutral organization	273	5.77 (0.84)	–5.21	< 0.001
	Happiness promoting organization	590	6.08 (0.69)		
Career	Neutral organization	273	5.20 (0.92)	–5.25	< 0.001
	Happiness promoting organization	590	5.55 (0.90)		
Innovation	Neutral organization	273	5.48 (0.94)	–5.63	< 0.001
	Happiness promoting organization	590	5.85 (0.81)		
Team	Neutral organization	273	5.58 (0.85)	–5.48	< 0.001
	Happiness promoting organization	590	5.91 (0.73)		
Organization	Neutral organization	273	5.72 (0.84)	–4.93	< 0.001
	Happiness promoting organization	590	6.02 (0.76)		

We also examined the differences in perceptions of happy colleagues’ performance between participants who chose an organization with happy employees and those who opted for an organization with neutral employees (scenario 2). Table 4 presents the *t*-test results. Overall, participants selecting an organization with happy colleagues had more positive views of their performance in all dimensions compared to those who chose an organization with neither happy nor unhappy employees. These associations between choices in the employment scenarios and perceptions of happy colleagues’ performance validate the measures of performance perceptions.

**TABLE 4:** The relationship between employment decisions in scenario 2 and happy colleagues’ job performance dimensions.

Index	Group	N	M(SD)	*T*	*P*-value
Job	Organization with neither happy nor unhappy employees’ colleagues	364	5.82 (0.80)	–5.10	<0.001
	Organization with happy colleagues	499	6.09 (0.69)		
Career	Organization with neither happy nor unhappy employees’ colleagues	364	5.24 (0.90)	–5.55	<0.001
	Organization with happy colleagues	499	5.59 (0.90)		
Innovation	Organization with neither happy nor unhappy employees’ colleagues	364	5.54 (0.90)	–5.40	<0.001
	Organization with happy colleagues	499	5.87 (0.82)		
Team	Organization with neither happy nor unhappy employees’ colleagues	364	5.63 (0.79)	–5.82	<0.001
	Organization with happy colleagues	499	5.94 (0.76)		
Organization	Organization with neither happy nor unhappy employees’ colleagues	364	5.75 (0.82)	–5.52	<0.001
	Organization with happy colleagues	499	6.05 (0.75)		

### Affective attitudes and happiness as predictors of performance perception

To examine the intercorrelations of the study variables and socio-demographic factors, Pearson’s correlation analysis was conducted. Results are reported in the online Supplementary section 7. The findings show a positive relationship between both trust and perceptions of happy colleagues’ performance, as well as between positive affect and performance perceptions. In contrast, negative affect toward happy colleagues is negatively correlated with perceptions of their performance. Additionally, positive correlations were observed between life meaning, valuing happiness, and perceptions of happy colleagues’ performance. Global life evaluation positively correlated with positive affect and affective trust in happy colleagues.

Multiple linear regressions were conducted to investigate the roles of affective attitudes and happiness variables in predicting perceptions of happy colleagues’ performance across five job performance dimensions: job, career, innovation, team, and organization. The independent variables included four dimensions of affective attitudes toward happy colleagues—affective trust, cognitive trust, negative affect, and positive affect—and five happiness and wellbeing variables: global life evaluation, meaning, valuing happiness, job satisfaction, and job meaning. Out of the socio-demographic variables, we selected age, gender (1 = female), education, job tenure and job position (1 = manager) to be included as control variables due to their potential effects on the dependent variables identified in the literature (Iverson et al., 1998; Ghasemy and Frömbling, 2023; Wright and Staw, 1999).

Table 5 displays the regression analysis. Affective attitudes toward happy colleagues, particularly positive affect and cognitive trust, significantly predicted perceptions of their performance. Among the happiness variables, valuing happiness significantly predicted perceptions of happy colleagues’ performance, while job meaning positively predicted perceptions of the career dimension. In contrast, global life evaluation, life meaning, and job satisfaction did not predict perceptions of happy colleagues’ performance, contradicting hypothesis 2. Finally, education negatively predicted perceptions of happy colleagues’ performance for the job dimension, whereas job tenure positively predicted perceptions of performance for the career dimension.

**TABLE 5:** Linear regressions for affective attitudes and happiness as predictors of performance perception.

Variable	Job	Career	Innovation	Team	Organization
	B(SE)	B(SE)	B(SE)	B(SE)	B(SE)
**Affective attitudes**					
Affective trust	–0.00 (0.03)	0.08 (0.04)^a^	0.07 (0.03)^b^	0.11 (0.03)***	0.07 (0.03)*
Cognitive trust	0.26 (0.03)***	0.22 (0.05)***	0.28 (0.05)***	0.21 (0.04)***	0.27 (0.04)***
Negative affect	–0.06 (0.02)*	0.00 (0.03)	–0.04 (0.03)	–0.09 (0.03)**	–0.05 (0.03)
Positive affect	0.22 (0.03)***	0.20 (0.04)***	0.16 (0.04)***	0.18 (0.03)***	0.20 (0.04)***
**Happiness and wellbeing**					
Global life evaluation	–0.02 (0.01)	–0.03 (0.01)	–0.03 (0.01)	–0.01 (0.01)	–0.03 (0.01)
Meaning	0.00 (0.01)	0.00 (0.01)	0.01 (0.01)	0.00 (0.01)	0.00 (0.01)
Valuing happiness	0.06 (0.01)**	0.08 (0.02)**	0.10 (0.02)***	0.04 (.02)*	0.02 (0.02)
Job satisfaction	–0.01 (0.01)	–0.00 (0.01)	–0.00 (0.01)	0.00 (0.01)	–0.00 (0.01)
Job meaning	0.02 (.01)	0.03 (0.01)*	0.02 (0.01)	0.01 (0.01)	0.02 (0.01)
**Socio-demographics**					
Age	–0.00 (0.00)	–0.00 (0.00)	0.00 (0.00)	–0.00(0.00)	0.00 (0.00)
Gender (1 = Female)	0.08 (0.04)	–0.02 (0.05)	0.08 (0.05)	0.05 (0.05)	0.05 (0.04)
Job position (1 = Manager)	–0.04 (0.04)	–0.02 (0.05)	–0.03(0.05)	–0.02 (0.05)	0.02 (0.04)
Education	–0.04 (0.01)*	–0.03 (0.02)	0.00	–0.01 (0.02)	–0.01 (0.01)
Job tenure	0.00 (0.00)	0.01 (0.00)*	0.00 (0.00)	0.00 (0.00)	0.00 (0.00)
Constant	3.45 (0.27)***	2.61 (0.35)***	2.55 (0.32)***	3.30 (0.28)***	3.00 (0.29)***
Adjusted *R*^2^	0.35	0.23	0.29	0.34	0.31
F	33.53	19.82	26.11	33.01	29.32
*P*-value	< 0.001	<0.001	< 0.001	<0.001	< 0.001

***p <0.001 **p < 0.01 *p < 0.05. The VIFs for the regression range from 1.034 to 2.802, indicating no multicollinearity.

^a^ This value is marginally significant, with *p* = 0.058.

^b^ This value is marginally significant, with *p* = 0.053.

Overall, the regression analyses reveal that employees’ feelings and trust in their happy colleagues predict perceptions of their performance, which can also be interpreted as a contribution of positive affect and trust to a happiness halo effect. An indication of a happiness halo effect in perceptions of happy colleagues’ performance may also be predicted by the degree to which employees value happiness—employees who value happiness as a personal goal are more likely to perceive their happy colleagues as performing well in their jobs.

### Mediating role of affective attitudes in the relationship between happiness and performance perceptions

Mediation analyses were conducted to assess whether affective attitudes toward happy colleagues mediate the relationship between happiness and perceptions of those colleagues’ job performance. Independent variables included global life evaluation, life meaning, value attributed to happiness, job satisfaction, and job meaning, while the five dimensions of job performance were the dependent variables. Affective trust, cognitive trust, and negative and positive affect served as mediators. Control variables included participants’ age, gender (1 = female), job position (1 = managerial role), education, and job tenure. A total of 25 models were established.^5^

Tables 6–8 display the results of the mediation analysis. (Full mediation analysis results are presented in the online Supplementary sections 8–12). We identified small but significant indirect effects between global life evaluation and perceptions of happy colleagues performance, with positive affect and affective trust as mediators (except for the job dimension). These findings indicate that employees’ global life evaluation predicts their positive affect and affective trust in happy colleagues, which in turn predict their perceptions of colleagues’ performance across dimensions of career, innovation, team, and organization.

**TABLE 6:** Direct and indirect effects in the mediation of affective attitudes on the relationship between global life evaluation and perceptions of happy colleagues’ performance.

Pathway	Effect relationship	*B*	Bootstrap 95% CI	
			Lower	Upper
**Model 1: X = Global life evaluation, M1 = affective trust, M2 = Cognitive trust, M3 = Negative affect, M4 = positive affect, Y = Job**				
Global life evaluation → job	Direct effects (c’)	–0.005	–0.030	0.019
Global life evaluation → trust → job	Indirect effects (a1 × b1)	0.000	–0.003	0.005
Global life evaluation → cognitive trust → job	Indirect effects (a2 × b2)	0.004	–0.004	0.014
Global life evaluation → negative affect → job	Indirect effects (a3 × b3)	0.000	–0.002	0.002
Global life evaluation → positive affect → job	Indirect effects (a4 × b4)	0.012	0.002	0.023
**Model 2: X = Global life evaluation, M1 = affective trust, M2 = Cognitive trust, M3 = Negative affect, M4 = positive affect, Y = Career**				
Global life evaluation → career	Direct effects (c’)	0.000	–0.033	0.033
Global life evaluation → affective trust → career	Indirect effects (a1 × b1)	0.006	0.000^a^	0.014
Global life evaluation → cognitive trust → career	Indirect effects (a2 × b2)	0.004	–0.004	0.013
Global life evaluation → negative affect → career	Indirect effects (a3 × b3)	0.000	–0.001	0.001
Global life evaluation → positive affect → career	Indirect effects (a4 × b4)	0.012	0.002	0.023
**Model 3: X = Global life evaluation, M1 = affective trust, M2 = Cognitive trust, M3 = Negative affect, M4 = positive affect, Y = Innovation**				
Global life evaluation →innovation	Direct effects (c’)	0.006	–0.024	0.037
Global life evaluation → affective trust → innovation	Indirect effects (a1 × b1)	0.006	0.000^b^	0.013
Global life evaluation → cognitive trust → innovation	Indirect effects (a2 × b2)	0.005	–0.005	0.015
Global life evaluation → negative affect → innovation	Indirect effects (a3 × b3)	0.000	–0.002	0.002
Global life evaluation →positive affect → innovation	Indirect effects (a4 × b4)	0.009	0.002	0.019
**Model 4: X = Global life evaluation, M1 = affective trust, M2 = Cognitive trust, M3 = Negative affect, M4 = positive affect, Y = Team**				
Global life evaluation → team	Direct effects (c’)	0.005	–0.021	0.031
Global life evaluation → affective trust → team	Indirect effects (a1 × b1)	0.008	0.002	0.016
Global life evaluation → cognitive trust →team	Indirect effects (a2 × b2)	0.003	–0.003	0.011
Global life evaluation → negative affect →team	Indirect effects (a3 × b3)	–0.000	–0.004	0.003
Global life evaluation positive affect team	Indirect effects (a4 × b4)	0.010	0.002	0.021
**Model 5: X = Global life evaluation, M1 = affective trust, M2 = Cognitive trust, M3 = Negative affect, M4 = positive affect, Y = Organization**				
Global life evaluation → organization	Direct effects (c’)	–0.012	–0.039	0.014
Global life evaluation → affective trust → organization	Indirect effects (a1 × b1)	0.005	0.000^c^	0.012
Global life evaluation → cognitive trust → organization	Indirect effects (a2 × b2)	0.004	–0.004	0.014
Global life evaluation → negative affect → organization	Indirect effects (a3 × b3)	0.000	–0.002	0.002
Global life evaluation → positive affect → organization	Indirect effects (a4 × b4)	0.011	0.002	0.021

^a^ The full number is 0.0007.

^b^ The full number is 0.0006.

^c^ The full number is 0.0002.

**TABLE 7:** Direct and indirect effects in the mediation of affective attitudes on the relationship between life meaning and perceptions of happy colleagues’ performance.

Pathway	Effect relationship	*B*	Bootstrap 95% CI	
			Lower	Upper
**Model 1: X = Life meaning, M1 = affective trust, M2 = Cognitive trust, M3 = Negative affect, M4 = positive affect, Y = Job**				
Life meaning → Job	Direct effects (c’)	0.016	–0.003	0.035
Life meaning ? affective trust → job	Indirect effects (a1 × b1)	0.000	–0.005	0.005
Life meaning → cognitive trust → job	Indirect effects (a2 × b2)	0.019	0.011	0.029
Life meaning → negative affect → job	Indirect effects (a3 × b3)	0.003	0.000^a^	0.007
Life meaning → positive affect → job	Indirect effects (a4 × b4)	0.022	0.012	0.032
**Model 2: X = Life meaning, M1 = affective trust, M2 = Cognitive trust, M3 = Negative affect, M4 = positive affect, Y = Career**				
Life meaning → career	Direct effects (c’)	0.030	0.004	0.055
Life meaning → affective trust → career	Indirect effects (a1 × b1)	0.007	0.000^b^	0.015
Life meaning → cognitive trust → career	Indirect effects (a2 × b2)	0.016	0.007	0.028
Life meaning → negative affect → career	Indirect effects (a3 × b3)	–0.000	–0.005	0.004
Life meaning → positive affect → career	Indirect effects (a4 × b4)	0.021	0.010	0.032
**Model 3: X = Life meaning, M1 = affective trust, M2 = Cognitive trust, M3 = Negative affect, M4 = positive affect, Y = Innovation**				
Life meaning → innovation	Direct effects (c’)	0.034	0.011	0.058
Life meaning → Affective trust → innovation	Indirect effects (a1 × b1)	0.006	0.000^c^	0.014
Life meaning → cognitive trust → innovation	Indirect effects (a2 × b2)	0.021	0.012	0.032
Life meaning → negative affect → innovation	Indirect effects (a3 × b3)	0.002	–0.002	0.008
Life meaning → positive affect → innovation	Indirect effects (a4 × b4)	0.016	0.007	0.028
**Model 4: X = Life meaning, M1 = affective trust, M2 = Cognitive trust, M3 = Negative affect, M4 = positive affect, Y = Team**				
Life meaning → team	Direct effects (c’)	0.020	0.000^d^	0.040
Life meaning → affective trust → team	Indirect effects (a1 × b1)	0.009	0.003	0.017
Life meaning → cognitive trust → team	Indirect effects (a2 × b2)	0.015	0.008	0.024
Life meaning → negative affect → team	Indirect effects (a3 × b3)	0.005	0.001	0.011
Life meaning → positive affect → team	Indirect effects (a4 × b4)	0.019	0.009	0.031
**Model 5: X = Life meaning, M1 = affective trust, M2 = Cognitive trust, M3 = Negative affect, M4 = positive affect, Y = Organization**				
Life meaning → organization	Direct effects (c’)	0.011	–0.009	0.032
Life meaning → affective trust → organization	Indirect effects (a1 × b1)	0.006	–0.000	0.013
Life meaning → cognitive trust → organization	Indirect effects (a2 × b2)	0.020	0.012	0.030
Life meaning → negative affect → organization	Indirect effects (a3 × b3)	0.003	–0.000	0.008
Life meaning → positive affect → organization	Indirect effects (a4 × b4)	0.019	0.010	0.030

^a^ The full number is 0.0000.

^b^ The full number is 0.0003.

^c^ The full number is 0.0005.

^d^ The full number is 0.0008.

**TABLE 8:** Direct and indirect effects in the mediation of affective attitudes on the relationship between valuing happiness and perceptions of happy colleagues’ performance.

Pathway	Effect relationship	*B*	Bootstrap 95% CI	
			Lower	Upper
**Model 1: X = Valuing happiness, M1 = affective trust, M2 = Cognitive trust, M3 = Negative affect, M4 = positive affect, Y = Job**				
Valuing happiness → job	Direct effects (c’)	0.064	0.030	0.098
Valuing happiness → affective trust → job	Indirect effects (a1 × b1)	–0.000	–0.011	0.009
Valuing happiness → cognitive trust →job	Indirect effects (a2 × b2)	0.035	0.020	0.054
Valuing happiness → negative affect → job	Indirect effects (a3 × b3)	0.003	0.000^a^	0.009
Valuing happiness → positive affect → job	Indirect effects (a4 × b4)	0.040	0.022	0.061
**Model 2: X = Valuing happiness, M1 = affective trust, M2 = Cognitive trust, M3 = Negative affect, M4 = positive affect, Y = Career**				
Valuing happiness → career	Direct effects (c’)	0.095	0.050	0.140
Valuing happiness →affective trust → career	Indirect effects (a1 × b1)	0.013	–0.000	0.029
Valuing happiness → cognitive trust → career	Indirect effects (a2 × b2)	0.030	0.013	0.050
Valuing happiness → negative affect → career	Indirect effects (a3 × b3)	0.000	–0.005	0.005
Valuing happiness → positive affect → career	Indirect effects (a4 × b4)	0.038	0.018	0.060
**Model 3: X = Valuing happiness, M1 = affective trust, M2 = Cognitive trust, M3 = Negative affect, M4 = positive affect, Y = Innovation**				
Valuing happiness → innovation	Direct effects (c’)	0.112	0.071	0.153
Valuing happiness → affective trust → innovation	Indirect effects (a1 × b1)	0.012	–0.000	0.027
Valuing happiness → cognitive trust → innovation	Indirect effects (a2 × b2)	0.038	0.021	0.059
Valuing happiness → negative affect → innovation	Indirect effects (a3 × b3)	0.003	–0.001	0.010
Valuing happiness → positive affect → innovation	Indirect effects (a4 × b4)	0.029	0.011	0.050
**Model 4: X = Valuing happiness, M1 = affective trust, M2 = Cognitive trust, M3 = Negative affect, M4 = positive affect, Y = Team**				
Valuing happiness → team	Direct effects (c’)	0.052	0.017	0.088
Valuing happiness → affective trust → team	Indirect effects (a1 × b1)	0.019	0.006	0.034
Valuing happiness → cognitive trust → team	Indirect effects (a2 × b2)	0.027	0.014	0.044
Valuing happiness → negative affect → team	Indirect effects (a3 × b3)	0.006	0.000^b^	0.014
Valuing happiness → positive affect → team	Indirect effects (a4 × b4)	0.035	0.017	0.057
**Model 5: X = Valuing happiness, M1 = affective trust, M2 = Cognitive trust, M3 = Negative affect, M4 = positive affect, Y = Organization**				
Valuing happiness → organization	Direct effects (c’)	0.024	–0.012	0.061
Valuing happiness → affective trust → organization	Indirect effects (a1 × b1)	0.012	–0.000	0.026
Valuing happiness → cognitive trust → organization	Indirect effects (a2 × b2)	0.037	0.021	0.055
Valuing happiness → negative affect → organization	Indirect effects (a3 × b3)	0.003	–0.000	0.009
Valuing happiness → Positive affect →Organization	Indirect effects (a4 × b4)	0.037	0.019	0.058

^a^ The full number is 0.0000.

^b^ The full number is 0.0007.

Additionally, small but significant indirect effects were found between life meaning and perceptions of happy colleagues’ performance, with positive affect, and affective (except for the organization dimension) and cognitive trust as mediators. Accordingly, positive affect and cognitive trust were found to mediate the relationship between valuing happiness and perceptions of happy colleagues’ performance. Negative affect and affective trust were identified as mediators in both of these relationships—life meaning and valuing happiness—with perceptions of performance on the team dimension. Overall, these findings indicate that employees’ sense of life meaning, and the value they assign to happiness predict their trust and affect toward their happy colleagues, which in turn predict their perceptions of the colleagues’ job performance. Similar results were found for job satisfaction and meaning. These findings are consistent with hypothesis 3, demonstrating that affective attitudes and trust in happy colleagues mediate the relationship between employees’ own happiness and their perceptions of these colleagues’ job performance.

We found no significant direct effects between global life evaluation and perceptions of performance in any of the all five dimensions, nor between life meaning and perceptions of performance on the job and organization dimensions, indicating that there are only indirect mediations (Zhao et al., 2010). Conversely, we found significant direct effects between life meaning and perceptions of performance on the career, innovation, and team dimensions, as well as between valuing happiness and perceptions of performance in all dimensions, indicating complementary mediations (Zhao et al., 2010).

### The moderating effect of happiness on the relationship between valuing happiness and perceptions of happy colleagues’ performance

Moderation analyses were conducted to explore the effect of happiness on the relationship between valuing happiness and perceptions of colleagues’ performance. We used the PROCESS macro program (version 4.2) (Hayes, 2022) [model 1], including bootstrapping with 5,000 resamples and 95% confidence intervals. Valuing happiness served as the independent variable, while perceptions of colleagues’ performance across the five dimensions were the dependent variables. Global life evaluation was included as the moderator, and age, gender, job position, education, and job tenure were included as covariates.

Table 9 presents the moderation results. In contrast to hypothesis 4, no significant interaction effects were found in any of the job performance domains.

**TABLE 9:** Moderation analyses results.

Variable	Job	Career	Innovation	Team	Organization
	B(SE)	B(SE)	B(SE)	B(SE)	B(SE)
Valuing happiness	0.15 (0.02)***	0.18 (0.02)***	0.20 (0.02)***	0.14 (0.02)***	0.12 (0.02)***
Global life evaluation	–0.02 (0.01)	–0.01 (0.01)	–0.01 (0.01)	–0.00 (0.01)	–0.01(.01)
Valuing happiness × global life evaluation	–0.00 (0.01)	0.02 (0.01)^a^	0.00 (0.01)	0.00 (0.01)	–0.00 (0.01)
Age	–0.00 (0.00)*	–0.00 (0.00)**	–0.00 (0.00)	–0.00 (0.00)**	–0.00 (0.00)
Gender (1 = Female)	0.11 (0.05)	0.01 (0.06)	0.12 (0.05)*	0.09 (0.05)	0.08 (0.05)
Job position (1 = Manager)	–0.01 (0.05)	0.01 (0.06)	0.00 (0.06)	–0.00 (0.05)	0.04 (0.05)
Education	–0.04 (.01)	–0.02 (0.02)	–0.00 (0.02)	–0.01 (0.01)	–0.01 (0.01)
Job tenure	0.00 (0.00)	0.01 (0.00)**	0.00 (0.00)	0.00 (0.00)	0.00 (0.00)
*R* ^2^	0.08	0.08	0.08	0.06	0.04
F	9.35	9.55	10.35	7.82	4.56
*P*-value	< 0.001	< 0.001	< 0.001	< 0.001	< 0.001

***p < 0.001 **p < 0.01 *p < 0.05.

^a^ This value is marginally significant, with *p* = 0.056.

## Discussion

This study examined the happiness halo effect and its impact on employees’ perceptions of happy colleagues, alongside the relationship between these perceptions and employees’ own happiness. The findings indicate that the happiness halo influences how employees perceive their happy colleagues, as well as the relationship of these perceptions to their own happiness.

Firstly, the study demonstrated an overall positive perception of happy colleagues in all job performance dimensions. Most participants stated that the happier their colleagues, the more they perform the tasks in their job description, advance in their careers, demonstrate innovation, collaborate with coworkers, and contribute to the organization. This pattern supports the happiness halo effect, whereby happiness can lead to positive evaluation in unrelated traits (Walsh et al., 2018). While some of these evaluations may reflect true differences in performance, the pervasiveness and consistency of the positive ratings suggest a perceptual bias driven by perceived happiness. Furthermore, this pattern aligns with previous research showing favorable evaluations of happy and positive employees by supervisors (Cropanzano and Wright, 1999; Hosie et al., 2012; Staw et al., 1994), as well as more favorable evaluations of employees with positive affectivity—though not specifically happy employees – by their colleagues (Ghasemy and Frömbling, 2023; Iverson et al., 1998; Staw and Barsade, 1993). Overall, this finding suggests that happy employees are generally perceived positively by their peers, which can contribute to a more positive work atmosphere.

However, while happy employees are generally perceived positively by their colleagues, the full picture might be more complex. This study found that colleagues’ own happiness, affective attitudes, and trust in happy employees might play a crucial role. Although the direct relationship between employees’ happiness and perceptions of their happy colleagues was not confirmed, an indirect relationship emerged, mediated by employees’ affective attitudes and trust in happy colleagues. Specifically, employees’ happiness predicted their positive affect and trust in their happy colleagues, which in turn predicted positive perceptions of their performance. Other aspects related to happiness, particularly a sense of life meaning and valuing happiness, were directly related to positive perceptions of happy colleagues’ performance across several dimensions, with further indirect effects through trust and positive affect. Although these effects were generally small in magnitude, the pathways described were consistent across the different models, suggesting that these evaluations are shaped not only by employees’ happiness itself, but also by their own affective attitudes and trust toward their happy colleagues.

These findings offer a more nuanced picture of how happy employees are perceived by their colleagues, suggesting that their colleagues’ own happiness may play a role in these perceptions. While employee happiness generally contributes to positive evaluations, it may also lead to less favorable reactions among unhappy colleagues. This aligns with the notion that others’ happiness can evoke varied feelings (Clark and Monin, 2014), and that unhappy individuals may experience negative emotions, or at least less positive ones, when observing happiness in others (Hamman, 2015; Uchida and Kitayama, 2009), potentially reducing the halo effect (Forgas, 2011). It is indeed possible, as suggested by the theory that happiness is relative (Easterlin, 1974; Veenhoven, 1991) and the social comparison theory (Festinger, 1954), that observing others’ happiness may trigger self-comparisons and feelings of inadequacy among unhappy individuals, leading to less favorable attitudes toward happy colleagues in other areas (Cohen-Charash and Mueller, 2007; Yada and Ikegami, 2014). If this is the case, then happy employees may not always have a positive impact on their colleagues, and unhappy employees may prefer working with less happy colleagues in order to protect their self-concept and avoid distress from social comparison (Wood, 1989). Taken together, these findings suggest that the happiness halo effect is not universal. As highlighted in this research, happiness may not be for everyone: being surrounded by happy colleagues, or even working in a generally happy environment, does not always produce positive outcomes and may even trigger negative affect among some employees.

Following this notion, we hypothesized that employees’ happiness and the importance they place on happiness would interact to influence their perceptions of happy colleagues’ performance. Specifically, we predicted that the more employees value happiness as a central life goal, the stronger the relationship between their happiness and their perceptions of happy colleagues. Since placing high importance on happiness might reduce happiness levels when it is perceived as attainable (Mauss et al., 2011), we predicted that unhappy employees who value happiness highly would have less positive perceptions of happy colleagues, whereas happy employees who value happiness would hold the most positive views. This assumption was not supported by the study.

Furthermore, teamwork emerged as a significant job performance dimension in this study, reflecting collaborative relationships among coworkers. Notably, perceptions of happy colleagues’ performance in teamwork were uniquely predicted by all aspects of trust and affective attitudes, including negative affect. Moreover, negative affect mediated the relationship between happiness and the teamwork dimension. These findings highlight a distinction between perceptions of social relationships at work and perceptions of performance in other domains, suggesting that perceptions of performance in teamwork may involve an emotional rather than cognitive appraisal, linking to various facets of affective attitudes and trust.

Overall, this study offers valuable insights into the happiness halo effect in employees’ perceptions of their colleagues, showing that while this effect does exist in the context of coworker relationships, the employees’ own happiness plays a role in shaping these perceptions. Although happy employees are generally viewed positively by their colleagues, unhappy colleagues may hold less favorable views. Perceptions of colleagues can have an effect on employees’ satisfaction and performance (Babin and Boles, 1996; Borgogni et al., 2010) and sometimes are part of employee evaluations (Masanja and Rweyemamu, 2020). These findings have practical and social implications for organizations and societies.

First, these findings have direct implications for different organizational aspects, like employee evaluations and team functioning. The tendency to positively evaluate happy employees can have implications on peer evaluations conducted in the organization, both informal and formal ones, such as 360-degree feedback (Masanja and Rweyemamu, 2020). Understanding this effect allows organizations to interpret evaluation results more accurately and to be mindful of potential biases in performance assessments. Moreover, when forming teams at work, organizations should consider employees’ happiness levels to foster collaboration and avoid negative social comparisons that could adversely affect less happy team members.

Second, these findings inform broader organizational policies and practices. The presence of happy employees can enhance overall employee attitudes and performance and contribute to a positive organizational climate. Therefore, organizations are encouraged to implement initiatives that strengthen employee happiness, such as improving work–life balance, fostering a positive climate, and enhancing social cohesion among employees. Promoting policies that enhance workplace happiness can attract and retain employees who value wellbeing, thereby reinforcing a positive organizational climate and fostering positive relations among the coworkers. However, while fostering a positive work environment and happiness among employees, organizations should also be aware of the potential negative effects on certain employees, especially less happy ones. The policies discussed above should simultaneously address the needs of these employees by providing emotional support, sensitivity, and inclusive practices that recognize individual differences in happiness. This understanding is the first step in addressing the needs of a broader range of employees, including unhappy ones, to enhance overall wellbeing, while recognizing that happiness, and the push toward it, may not be for all.

Finally, in an era prioritizing happiness, social policies that promote overall wellbeing can encourage positive experiences and attitudes, but they should also recognize that not all individuals respond equally to happiness-promoting initiatives. Inclusive and sensitive approaches are necessary to ensure that interventions support all members of society, respecting individual differences and providing appropriate support for those who may experience negative effect.

Despite the insights of this study, several limitations should be acknowledged, which future research could address. This study was conducted among people employed in the United States and may be influenced by cultural bias. For example, the individualism prevalent in American work culture (Hofstede, 1980) could lead to differing perceptions of happy coworkers from those in more collectivist environments. Additionally, the experience of happiness itself varies between different cultures (Cheng S. L. et al., 2025). Future research could address this limitation by using a more heterogeneous sample that includes employees from diverse countries and work cultures.

Additionally, due to its cross-sectional design, in which data were self-reported by participants at a single point in time, this study is exposed to the potential risk of common method variance (CMV). Such potential bias can make it difficult to draw causal inferences from the findings and to be certain that the results do not reflect systematic measurement error (Podsakoff et al., 2003). Podsakoff et al. (2003) suggested several practical strategies to reduce the risk of CMV, which could be implemented in future research. For example, temporal separation of measurements could reduce both temporary biases and those arising from completing all measures at once, providing more stable and reliable data. Additionally, collecting data from multiple sources could minimize systematic error resulting from reliance on a single rater. For instance, the happiness scale could be completed not only by employees, but also by their colleagues or even their partners. Implementing these strategies in future studies would strengthen causal inferences and provide a more robust understanding of the relationships examined in this research.

Moreover, this study utilized happiness measures based on previous studies (Barokas et al., 2022; Helliwell et al., 2020), including global life evaluation, life meaning, and positive and negative affectivity. However, given the complexity of happiness (Yang et al., 2021), it is possible that additional measures could provide a more comprehensive understanding. For instance, some studies assess happiness in specific life areas (Bojanowska and Zalewska, 2016), or through a direct general question about how happy the respondent is (Abdel-Khalek, 2006). In this study, we employed aspects of the RBPS developed by Welbourne et al. (1998) to measure perceptions of job performance across job, career, innovation, team, and organization dimensions. However, RBPS is only one of many scales measuring job performance, each reflecting different approaches (Ramos-Villagrasa et al., 2019). Therefore, future research could integrate additional measures of happiness and perceived job performance to enhance understanding of how happy employees are viewed by their colleagues, and how this relates to their own happiness.

## Data Availability

The datasets presented in this study can be found at: https://osf.io/zfm6e/?view_only=cda1546ecb55411bbff7d13c3c244ca6.

## Footnotes

1 The questionnaire and methodology for this study were approved by the University Research Ethics Committee.

2 The questions were: (1) For technical reasons, choose the option “somewhat more”; (2) For technical reasons, choose the option “neither less nor more.”

3 Helliwell et al. (2020), page 19 and Table 2.1, page 21.

4 Helliwell et al. (2020), Table 2.1, page 21 and Technical Box 1, page 22.

5 Due to the small magnitude of the observed effects, and for increased precision and accuracy, statistical values in the mediation analyses are reported to three decimal places.
