# Supplementary material for: Is happiness for all? The happiness halo effect on coworkers’ perceptions
Source: Front Psychol. 2026 Jan 6;16:1653843. doi: 10.3389/fpsyg.2025.1653843 (PMC12815774; doi:10.3389/fpsyg.2025.1653843)
Supplement: Supplementary file 1 [file Supplementary_file_1.docx]

**Online Supplement**

**Section 1: Perceptions of happy colleagues’** **job performance scale**

For each statement you will be required to rate various aspects of happy employees’ performance on a scale from **much less** to **much more**. Please respond in the context of employees working in your specific organization with whom you have daily contact.

|  | Much less | Less | Somewhat less | Neither less nor more | Somewhat more | More | Much more |
| --- | --- | --- | --- | --- | --- | --- | --- |
| 1. The happier employees are, the **less/more** they satisfy the quantity of work output that is officially required. | ☐ | ☐ | ☐ | ☐ | ☐ | ☐ | ☐ |
| 2. The happier employees are, the **less/more** they satisfy the quality of work output that is officially required. | ☐ | ☐ | ☐ | ☐ | ☐ | ☐ | ☐ |
| 3. The happier employees are, the **less/more** they perform their work accurately. | ☐ | ☐ | ☐ | ☐ | ☐ | ☐ | ☐ |
| 4. The happier employees are, the **less/more** they provide good customer service (internal & external). | ☐ | ☐ | ☐ | ☐ | ☐ | ☐ | ☐ |
| 5. The happier employees are, the **less/more** they obtain personal career goals. | ☐ | ☐ | ☐ | ☐ | ☐ | ☐ | ☐ |
| 6. The happier employees are, the **less/more** they develop skills needed for their future career. | ☐ | ☐ | ☐ | ☐ | ☐ | ☐ | ☐ |
| 7. The happier employees are, the **less/more** they make progress in their career. | ☐ | ☐ | ☐ | ☐ | ☐ | ☐ | ☐ |
| 8. The happier employees are, the **less/more** they seek out career opportunities. | ☐ | ☐ | ☐ | ☐ | ☐ | ☐ | ☐ |
| 9. The happier employees are, the **less/more** they come up with new ideas. | ☐ | ☐ | ☐ | ☐ | ☐ | ☐ | ☐ |
| 10. The happier employees are, the **less/more** they work to implement new ideas. | ☐ | ☐ | ☐ | ☐ | ☐ | ☐ | ☐ |
| 11. The happier employees are, the **less/more** they find improved ways to do things. | ☐ | ☐ | ☐ | ☐ | ☐ | ☐ | ☐ |
| 12. The happier employees are, the **less/more** they create better processes and routines. | ☐ | ☐ | ☐ | ☐ | ☐ | ☐ | ☐ |
| 13. The happier employees are, the **less/more** they work as part of a team or work group. | ☐ | ☐ | ☐ | ☐ | ☐ | ☐ | ☐ |
| 14. The happier employees are, the **less/more** they seek information from others in their work group. | ☐ | ☐ | ☐ | ☐ | ☐ | ☐ | ☐ |
| 15. The happier employees are, the **less/more** they make sure that the work group succeeds. | ☐ | ☐ | ☐ | ☐ | ☐ | ☐ | ☐ |
| 16. The happier employees are, the **less/more** they respond to the needs of others in their work group. | ☐ | ☐ | ☐ | ☐ | ☐ | ☐ | ☐ |
| 17. The happier employees are, the **less/more** they do things to help others when it’s not part of their job. | ☐ | ☐ | ☐ | ☐ | ☐ | ☐ | ☐ |
| 18. The happier employees are, the **less/more** they work for the overall good of the company. | ☐ | ☐ | ☐ | ☐ | ☐ | ☐ | ☐ |
| 19. The happier employees are, the **less/more** they do things that promote the company. | ☐ | ☐ | ☐ | ☐ | ☐ | ☐ | ☐ |
| 20. The happier employees are, the **less/more** they help so that the company is a good place to be workplace. | ☐ | ☐ | ☐ | ☐ | ☐ | ☐ | ☐ |

The measures:
Job – items 1-4
Career: items 5-8
Innovation: items 9-12
Team: items 13-16
Organization: items 17-20

**Section 2: Affective attitudes towards happy colleagues scale**

The following statements relate to the employees currently employed within your workplace.

For each statement you will be required to rate your attitudes towards happy employees on a scale from **much less** to **much more**. Please respond in the context of employees working in your specific organization with whom you have daily contact.

|  | Much less | Less | Somewhat less | Neither less nor more | Somewhat more | More | Much more |
| --- | --- | --- | --- | --- | --- | --- | --- |
| 1. The happier employees in my workplace are, the **less/more** I would share with them my most outlandish ideas and hopes. | ☐ | ☐ | ☐ | ☐ | ☐ | ☐ | ☐ |
| 2. The happier employees in my workplace are, the **less/more** I would talk with them about difficulties I am having at work. | ☐ | ☐ | ☐ | ☐ | ☐ | ☐ | ☐ |
| 3. The happier employees in my workplace are, the **less/more** I am willing to admit my worst mistakes to them. | ☐ | ☐ | ☐ | ☐ | ☐ | ☐ | ☐ |
| 4. The happier employees in my workplace are, the **less/more** I would rely on them for support when I need it. | ☐ | ☐ | ☐ | ☐ | ☐ | ☐ | ☐ |
| 5. The happier employees in my workplace are, the **less/more** I would reveal information to them that I don’t want others to know. | ☐ | ☐ | ☐ | ☐ | ☐ | ☐ | ☐ |
| 6. The happier employees in my workplace are, the **less/more** I would like to take their advice about work. | ☐ | ☐ | ☐ | ☐ | ☐ | ☐ | ☐ |
| 7. The happier employees in my workplace are, the **less/more** I would rely on them to follow through on commitments. | ☐ | ☐ | ☐ | ☐ | ☐ | ☐ | ☐ |
| 8. The happier employees in my workplace are, the **less/more** I would assume their work is done properly if I needed to use it. | ☐ | ☐ | ☐ | ☐ | ☐ | ☐ | ☐ |
| 9. The happier employees in my workplace are, the **less/more** I would feel comfortable having them in a critical role in my team. | ☐ | ☐ | ☐ | ☐ | ☐ | ☐ | ☐ |
| 10. The happier employees in my workplace are, the **less/more** I would feel uneasy if I needed to depend on their ability. | ☐ | ☐ | ☐ | ☐ | ☐ | ☐ | ☐ |
| 11. The happier employees in my workplace are, the **less/more** I would assume that they have a good reason if they show up late to a meeting. | ☐ | ☐ | ☐ | ☐ | ☐ | ☐ | ☐ |
| 12. Somehow it appears **less/more** fair that some employees in my workplace are happier than others. | ☐ | ☐ | ☐ | ☐ | ☐ | ☐ | ☐ |
| 13. The happier employees in my workplace are, the **less/more** I feel contempt towards them. | ☐ | ☐ | ☐ | ☐ | ☐ | ☐ | ☐ |
| 14. The bitter truth is that the happier employees in my workplace are, the **less/more** I generally feel inferior to them. | ☐ | ☐ | ☐ | ☐ | ☐ | ☐ | ☐ |
| 15. The happier employees in my workplace are, the **less/more** they make me feel tense. | ☐ | ☐ | ☐ | ☐ | ☐ | ☐ | ☐ |
| 16. The happier employees in my workplace are, the **less/more** I feel relatively inadequate. | ☐ | ☐ | ☐ | ☐ | ☐ | ☐ | ☐ |
| 17. The happier employees in my workplace are, the **less/more** I feel pity towards them. | ☐ | ☐ | ☐ | ☐ | ☐ | ☐ | ☐ |
| 18. The happier employees in my workplace are, the **less/more** I feel disgusted by them. | ☐ | ☐ | ☐ | ☐ | ☐ | ☐ | ☐ |
| 19. The happier employees in my workplace are, the **less/more** I feel stress thinking about them. | ☐ | ☐ | ☐ | ☐ | ☐ | ☐ | ☐ |
| 20. The happier employees in my workplace are, the **less/more** I feel close to them without knowing them. | ☐ | ☐ | ☐ | ☐ | ☐ | ☐ | ☐ |
| 21. Frankly, the happier employees in my workplace are, the **less/more** their success makes me resent them. | ☐ | ☐ | ☐ | ☐ | ☐ | ☐ | ☐ |
| 22. The happier employees in my workplace are, the **less/more** I feel repulsed by them. | ☐ | ☐ | ☐ | ☐ | ☐ | ☐ | ☐ |
| 23. The happier employees in my workplace are, the **less/more** I feel apprehensive towards them. | ☐ | ☐ | ☐ | ☐ | ☐ | ☐ | ☐ |
| 24. The happier an employee in my workplace is, the **less/more** I like them. | ☐ | ☐ | ☐ | ☐ | ☐ | ☐ | ☐ |
| 25. The happier employees in my workplace are, the **less/more** I get along well with them. | ☐ | ☐ | ☐ | ☐ | ☐ | ☐ | ☐ |
| 26. The happier employees in my workplace are, the **less/more** working with them is a pleasure. | ☐ | ☐ | ☐ | ☐ | ☐ | ☐ | ☐ |
| 27. The happier employees in my workplace are, the **less/more** I think they would make a good friend. | ☐ | ☐ | ☐ | ☐ | ☐ | ☐ | ☐ |

The measures:
Affective trust: items 1-5

Cognitive trust: items 6-11 (10 reversed)

Negative affect: items 12-23 (20 reversed)

Positive affect: Items 24-27

**Section 3: Happiness scale**

**Global life evaluation**

Imagine a ladder with rungs numbered from 0 at the bottom to 10 at the top. The top of the ladder expresses the best possible life for you, and the bottom of the ladder expresses the worst possible life for you. On which rung of the ladder are you at this stage of your life?

| Worst possible life |  |  |  |  |  |  |  |  |  | Best possible life |
| --- | --- | --- | --- | --- | --- | --- | --- | --- | --- | --- |
| 0 | 1 | 2 | 3 | 4 | 5 | 6 | 7 | 8 | 9 | 10 |

**Affective dimension**

Please provide below details that are related to your emotions yesterday. For each question, please indicate the level of the associated feelings by choosing the number that represents your feeling from 0-10, where: 0 - Not at all, 10 - Completely

| Statement | Not at all |  |  |  |  |  |  |  |  |  | Completely |
| --- | --- | --- | --- | --- | --- | --- | --- | --- | --- | --- | --- |
|  | 0 | 1 | 2 | 3 | 4 | 5 | 6 | 7 | 8 | 9 | 10 |
| Did you experience enjoyment during a lot of the day yesterday? | 0 | 1 | 2 | 3 | 4 | 5 | 6 | 7 | 8 | 9 | 10 |
| Did you experience exaltation during a lot of the day yesterday? | 0 | 1 | 2 | 3 | 4 | 5 | 6 | 7 | 8 | 9 | 10 |
| Did you smile or laughed during a lot of the day yesterday? | 0 | 1 | 2 | 3 | 4 | 5 | 6 | 7 | 8 | 9 | 10 |
| Did you experience concern during a lot of the day yesterday? | 0 | 1 | 2 | 3 | 4 | 5 | 6 | 7 | 8 | 9 | 10 |
| Did you experience sadness during a lot of the day yesterday? | 0 | 1 | 2 | 3 | 4 | 5 | 6 | 7 | 8 | 9 | 10 |
| Did you experience depression during a lot of the day yesterday? | 0 | 1 | 2 | 3 | 4 | 5 | 6 | 7 | 8 | 9 | 10 |
| Did you experience anger during a lot of the day yesterday? | 0 | 1 | 2 | 3 | 4 | 5 | 6 | 7 | 8 | 9 | 10 |

**Life meaning and purpose**

Overall, to what extent do you feel the things you do in your life are worthwhile?

| Not meaningful at all |  |  |  |  |  |  |  |  |  | Very meaningful |
| --- | --- | --- | --- | --- | --- | --- | --- | --- | --- | --- |
| 0 | 1 | 2 | 3 | 4 | 5 | 6 | 7 | 8 | 9 | 10 |

**Job satisfaction**

In general, to what extent are you satisfied with your workplace?

| Not at all |  |  |  |  |  |  |  |  |  | Very much |
| --- | --- | --- | --- | --- | --- | --- | --- | --- | --- | --- |
| 0 | 1 | 2 | 3 | 4 | 5 | 6 | 7 | 8 | 9 | 10 |

**Job meaning**

In general, to what extent do you feel that your job is meaningful?

| Not meaningful at all |  |  |  |  |  |  |  |  |  | Very meaningful |
| --- | --- | --- | --- | --- | --- | --- | --- | --- | --- | --- |
| 0 | 1 | 2 | 3 | 4 | 5 | 6 | 7 | 8 | 9 | 10 |

**Section 4: Valuing happiness**

1. How happy I am at any given moment says a lot about how worthwhile my life is

| Strongly disagree |  |  |  |  |  |  | Strongly agree |
| --- | --- | --- | --- | --- | --- | --- | --- |
| 0 | 1 | 2 | 3 | 4 | 5 | 6 | 7 |

1. Felling happy is extremely important to me.

| Strongly disagree |  |  |  |  |  |  | Strongly agree |
| --- | --- | --- | --- | --- | --- | --- | --- |
| 0 | 1 | 2 | 3 | 4 | 5 | 6 | 7 |

**Section 5: Employment scenarios**

**Scenario 1**

Say you have to choose between two organizations to be employed at within your field of expertise.
**Organization A** prioritizes promoting employee happiness as a significant objective. Consequently, the organization invests substantial efforts in preserving and enhancing the happiness of its employees. The organization employs a dedicated team responsible for advancing employee well-being, organizes social activities to boost employee happiness, and strives to increase employee job satisfaction. Working for this organization will result in a salary identical to your current one.
**Organization B** does not view employee happiness as a significant objective, and as a result, the organization does not undertake special efforts to promote it. Working for this organization will result in a salary 10% higher than your current one.
These two organizations require the same number of working hours and offer identical social conditions, except for the wage aspect.
There are two options:
**Option 1**: Working for organization A, which emphasizes promoting employee happiness without additional salary increments.
**Option 2**: Working for organization B, which does not prioritize employee happiness, offering a 10% salary increase.
Which option will you be more likely to choose?

| Option 2: Working for organization B, which does not prioritize employee happiness, with a 10% salary increase. | | | Option 1: Working in organization A, which emphasizes promoting employee happiness without additional salary increments | | |
| --- | --- | --- | --- | --- | --- |
| I am sure I will choose option 2 | Most likely, I will choose option 2 | Perhaps I will choose option 2 | Perhaps I will choose option 1 | Most likely, I will choose option 1 | I am sure I will choose option 1 |
| X | X | X | X | X | X |

**Scenario 2**

Say you have to choose between two organizations to be employed at within your field of expertise.
The employees in **organization A**are happy individuals. They maintain a positive outlook on life, and their overall life satisfaction is high. The employees’ happiness is reflected in their work. Working for this organization will result in a salary identical to your current one.
The employees in **organization B** are neither happy nor unhappy individuals; rather, their happiness is at an average level. Their outlook on life is predominantly neutral, and their overall life satisfaction is average. The employees’ average life satisfaction and happiness is reflected in their work. Working for this organization will result in a salary 10% higher than your current one.
These two organizations require the same number of working hours and offer identical social conditions, except for the wage aspect.
There are two options:
**Option 1**: Working for organization A, where the employees are happy individuals, without additional salary increments.
**Option 2**: Working for organization B, where the employees are neither happy nor unhappy individuals, with a 10% salary increase.
Which option will you be more likely to choose?

| Option 2: Working for organization B, where the employees are neither happy nor unhappy individuals, with a 10% salary increase | | | Option 1: Where the employees are happy individuals, without additional salary increments | | |
| --- | --- | --- | --- | --- | --- |
| I am sure I will choose option 2 | Most likely, I will choose option 2 | Perhaps I will choose option 2 | Perhaps I will choose option 1 | Most likely, I will choose option 1 | I am sure I will choose option 1 |
| X | X | X | X | X | X |

## **Section 6: Socio-demographic questions**

1. Age:
2. Gender: Male/ Female/ Other.
3. Are you in a relationship? Yes/ No
4. Number of children:
5. What is the highest education level you received? Some high school degree or less/ High school graduate/ Other post high school training/ Some college, no degree/ Associate degree/ Bachelor’s degree/ Master’s or Professional degree/ Doctoral degree.
6. Please indicate your general health status: Not good at all/ Not very good/ Average/ Good/ Very good.
7. To what extent do you consider yourself religious: Not religious at all/ Not very religious/ Somewhat religious/ Very religious.
8. What is your annual income? Under $25,000/ $25,000 to $34,999/ $35,000 to $49,999/ $50,000 to $74,999/ $75,000 to $99,999/ $100,000 to $124,900/ $125,000 to $149,999/ $150,000 or more
9. How satisfied you are with the financial resources at your disposal? 1 Not satisfied at all/ 2/ 3/ 4/ 5/ 6/ 7 Very satisfied
10. What is your current role/position? Executive (e.g., CEO, President)/ Upper management (e.g., VP, Director)/ Middle management (e.g., manager, supervisor)/ Professional staff (e.g., manager, supervisor)/ Technical staff (e.g., maintenance professional)/ Administrative staff/ Other
11. How long have you been working at your current job?
12. How many times per week are you required to come to work? (If you do not come to the office, please write 0)

**Section 7: Intercorrelations for study variables and sociodemographic factors**

| Variable | 1 | 2 | 3 | 4 | 5 | 6 | 7 | 8 | 9 | 10 | 11 | 12 | 13 | | 14 | | 15 | | 16 | | | 17 | | | 18 | | | 19 | | 20 | | | 21 | |
| --- | --- | --- | --- | --- | --- | --- | --- | --- | --- | --- | --- | --- | --- | --- | --- | --- | --- | --- | --- | --- | --- | --- | --- | --- | --- | --- | --- | --- | --- | --- | --- | --- | --- | --- |
| 1. Job | 1 |  |  |  |  |  |  |  |  |  |  |  |  | |  | |  | |  | | |  | | |  | | |  | |  | | |  | |
| 2. Career | .59*** | 1 |  |  |  |  |  |  |  |  |  |  |  | |  | |  | |  | | |  | | |  | | |  | |  | | |  | |
| 3. Innovation | .74*** | .66*** | 1 |  |  |  |  |  |  |  |  |  |  | |  | |  | |  | | |  | | |  | | |  | |  | | |  | |
| 4. Team | .75*** | .62*** | .77*** | 1 |  |  |  |  |  |  |  |  |  | |  | |  | |  | | |  | | |  | | |  | |  | | |  | |
| 5. Organization | .77*** | .54*** | .72*** | .77*** | 1 |  |  |  |  |  |  |  |  | |  | |  | |  | | |  | | |  | | |  | |  | | |  | |
| 6. Affective trust | .33*** | .34*** | .35*** | .41*** | .37*** | 1 |  |  |  |  |  |  |  | |  | |  | |  | | |  | | |  | | |  | |  | | |  | |
| 7. Cognitive trust | .52*** | .41*** | .48*** | .51*** | .51*** | .54*** | 1 |  |  |  |  |  |  | |  | |  | |  | | |  | | |  | | |  | |  | | |  | |
| 8. Negative affect | -.36*** | -.22*** | -.29*** | -.35*** | -.33*** | -.13*** | -.49*** | 1 |  |  |  |  |  | |  | |  | |  | | |  | | |  | | |  | |  | | |  | |
| 9. Positive affect | .52*** | .42*** | .45*** | .52*** | .50*** | .55*** | .67*** | -.48*** | 1 |  |  |  |  | |  | |  | |  | | |  | | |  | | |  | |  | | |  | |
| 10. Global life evaluation | -.00 | .02 | .04 | .04 | .01 | .12*** | .02 | .01 | .08* | 1 |  |  |  | |  | |  | |  | | |  | | |  | | |  | |  | | |  | |
| 11. Meaning | .16*** | .17*** | .20*** | .18*** | .16*** | .20*** | .19*** | -.14*** | .22*** | .42*** | 1 |  |  |  | |  | |  | |  | | |  | | |  | | |  | |  | |  |  |
| 12. Positive affectivity | .13*** | .21*** | .20*** | .20*** | .12*** | .22*** | .18*** | -.06* | .20*** | .36*** | .59*** | 1 |  |  | |  | |  | |  | | |  | | |  | | |  | |  | |  |  |
| 13. Negative affectivity | -.12*** | -.09** | -.07* | -.12*** | -.10** | -.09** | -.14*** | .20*** | -.16*** | -.22*** | -.40*** | -.40*** | 1 | |  | |  | |  | | |  | |  | | |  | | |  | |  | |  |
| 14. Value happiness | .23*** | .24*** | .28*** | .22*** | .18*** | .24*** | .21*** | -.07* | .25*** | .27*** | .39*** | .47*** | -.15*** | | 1 | |  | |  | | |  | |  | | |  | | |  | |  | |  |
| 15. Job satisfaction | .15*** | .18*** | .19*** | .18*** | .15*** | .23*** | .16*** | -.09** | .23*** | .38*** | .54*** | .49*** | -.32*** | | .38*** | | 1 | |  | | |  | |  | | |  | | |  | |  | |  |
| 16. Job meaning | .16*** | .20*** | .21*** | .19*** | .16*** | .22*** | .17*** | -.10** | .20*** | .38*** | .57*** | .47*** | -.21*** | | .37*** | | .77*** | | 1 | |  | | |  | | |  | | |  | |  | |  |
| 17. Age | -.06 | -.08* | -.05 | -.10** | -.02 | -.10** | -.11*** | -.02 | -.09** | .07* | .08* | -.04 | -.01 | | -.04 | | .06 | | .04 | | 1 | | |  | | |  | | |  | |  | |  |
| 18. Gender (1=Female) | .05 | -.02 | .04 | .03 | .03 | -.06* | .01 | -.07* | .01 | .00 | .02 | -.01 | .03 | | -.06 | | .01 | | .00 | | .00 | | | 1 | | |  | | |  | |  | |  |
| 19. Job position (1=Managerial Job) | -.01 | .02 | .02 | .00 | .03 | .07* | .03 | .02 | .01 | .24*** | .16*** | .14*** | -.04 | | .10** | | .21*** | | .22*** | | .07* | | | -.07* | | | 1 | | |  | |  | |  |
| 20. Education | -.10** | -.05 | -.02 | -.04 | -.04 | .00 | -.04 | .03 | -.02 | .29*** | .12*** | .08* | -.05 | | -.03 | | .11*** | | .18*** | | .01 | | | .02 | | | .24*** | | | 1 | |  | |  |
| 21. Job tenure | .02 | .04 | .00 | -.01 | .02 | .01 | -.01 | -.04 | -.01 | .08* | .11** | .03 | -.07* | | -.00 | | .09** | | .11** | | .48*** | | | -.07* | | | .16*** | | | .00 | | 1 | | |

***p<.001 **p<.01 *p < .05

**Section 8: Results of mediation analysis of the relationship between global life evaluation and perceptions of happy colleagues’ performance**

| **Pathway** | **Effect relationship** | **B** | | **Bootstrap 95% CI** | | | |
| --- | --- | --- | --- | --- | --- | --- | --- |
|  |  |  |  | **Lower** | | **Upper** | |
| **Model 1: X = Global life evaluation, M1 = Affective trust, M2 = Cognitive trust, M3 = Negative affect, M4 = Positive affect, Y = Job** | | | | | | | |
| Global life evaluation → Affective trust | Direct effects (a1) | .067 | | .031 | | .104 | |
| Global life evaluation → Cognitive trust | Direct effects (a2) | .017 | | -.015 | | .049 | |
| Global life evaluation → Negative affect | Direct effects (a3) | .000 | | -.038 | | .039 | |
| Global life evaluation → Positive affect | Direct effects (a4) | .052 | | .015 | | .089 | |
| Affective trust → Job | Direct effects (b1) | .007 | | -.052 | | .067 | |
| Cognitive trust → Job | Direct effects (b2) | .270 | | .193 | | .347 | |
| Negative affect → Job | Direct effects (b3) | -.054 | | -.108 | | -.001 | |
| Positive affect → Job | Direct effects (b4) | .236 | | .169 | | .303 | |
| Global life evaluation → Job | Direct effects (c’) | -.005 | | -.030 | | .019 | |
| Global life evaluation → Affective trust → Job | Indirect effects (a1×b1) | .000 | | -.003 | | .005 | |
| Global life evaluation → Cognitive trust → Job | Indirect effects (a2×b2) | .004 | | -.004 | | .014 | |
| Global life evaluation → Negative affect → Job | Indirect effects (a3×b3) | .000 | | -.002 | | .002 | |
| Global life evaluation → Positive affect → Job | Indirect effects (a4×b4) | .012 | | .002 | | .023 | |
| Global life evaluation → Job | Total effects (c) | .012 | | -.018 | | .042 | |
| Age → Job | Covariates | -.001 | | -.005 | | .003 | |
| Gender (1 = Female) → Job | Covariates | .071 | | -.012 | | .155 | |
| Job position (1 = Manager) → Job | Covariates | -.018 | | -.106 | | .070 | |
| Education → Job | Covariates | -.039 | | -.070 | | -.009 | |
| Job tenure → Job | Covariates | .005 | | -.002 | | .014 | |
| **Model 2: X = Global life evaluation, M1 = Affective trust, M2 = Cognitive trust, M3 = Negative affect, M4 = Positive affect, Y = Career** | | | | | | | |
| Global life evaluation → Affective trust | Direct effects (a1) | | .067 | | .031 | .104 | |
| Global life evaluation → Cognitive trust | Direct effects (a2) | | .017 | | -.015 | .049 | |
| Global life evaluation → Negative affect | Direct effects (a3) | | .000 | | -.038 | .039 | |
| Global life evaluation → Positive affect | Direct effects (a4) | | .052 | | .015 | .089 | |
| Affective trust → Career | Direct effects (b1) | | .097 | | .017 | .176 | |
| Cognitive trust → Career | Direct effects (b2) | | .230 | | .128 | .331 | |
| Negative affect → Career | Direct effects (b3) | | .003 | | -.066 | .074 | |
| Positive affect → Career | Direct effects (b4) | | .228 | | .140 | .317 | |
| Global life evaluation → Career | Direct effects (c’) | | .000 | | -.033 | .033 | |
| Global life evaluation → Affective trust → Career | Indirect effects (a1×b1) | | .006 | | .000^a^ | .014 | |
| Global life evaluation → Cognitive trust → Career | Indirect effects (a2×b2) | | .004 | | -.004 | .013 | |
| Global life evaluation → Negative affect → Career | Indirect effects (a3×b3) | | .000 | | -.001 | .001 | |
| Global life evaluation → Positive affect → Career | Indirect effects (a4×b4) | | .012 | | .002 | .023 | |
| Global life evaluation → Career | Total effects (c) | | .022 | | -.014 | .059 | |
| Age → Career | Covariates | | -.005 | | -.010 | .000 | |
| Gender (1 = Female) → Career | Covariates | | -.025 | | -.136 | .086 | |
| Job position (1 = Manager) | Covariates | | .013 | | -.103 | .130 | |
| Education → Career | Covariates | | -.029 | | -.069 | .010 | |
| Job tenure → Career | Covariates | | .011 | | .000^b^ | .022 | |
| **Model 3: X = Global life evaluation, M1 = Affective trust, M2 = Cognitive trust, M3 = Negative affect, M4 = Positive affect, Y = Innovation** | | | | | | | |
| Global life evaluation → Affective trust | Direct effects (a1) | | .067 | | .031 | .104 | |
| Global life evaluation → Cognitive trust | Direct effects (a2) | | .017 | | -.015 | .049 | |
| Global life evaluation → Negative affect | Direct effects (a3) | | .000 | | -.038 | .039 | |
| Global life evaluation → Positive affect | Direct effects (a4) | | .052 | | .015 | .089 | |
| Affective trust → Innovation | Direct effects (b1) | | .090 | | .018 | .163 | |
| Cognitive trust → Innovation | Direct effects (b2) | | .297 | | .203 | .390 | |
| Negative affect → Innovation | Direct effects (b3) | | -.041 | | -.105 | .023 | |
| Positive affect → Innovation | Direct effects (b4) | | .186 | | .104 | .267 | |
| Global life evaluation → Innovation | Direct effects (c’) | | .006 | | -.024 | .037 | |
| Global life evaluation → Affective trust → Innovation | Indirect effects (a1×b1) | | .006 | | .000^c^ | .013 | |
| Global life evaluation → Cognitive trust → Innovation | Indirect effects (a2×b2) | | .005 | | -.005 | .015 | |
| Global life evaluation → Negative affect → Innovation | Indirect effects (a3×b3) | | .000 | | -.002 | .002 | |
| Global life evaluation → Positive affect → Innovation | Indirect effects (a4×b4) | | .009 | | .002 | .019 | |
| Global life evaluation → Innovation | Total effects (c) | | .027 | | -.008 | .062 | |
| Age → Innovation | Covariates | | -.000 | | -.005 | .005 | |
| Gender (1 = Female) → Innovation | Covariates | | .071 | | -.030 | .173 | |
| Job position (1 = Manager) → Innovation | Covariates | | .007 | | -.099 | .115 | |
| Education → Innovation | Covariates | | -.006 | | -.042 | .030 | |
| Job tenure → Innovation | Covariates | | .001 | | -.008 | .011 | |
| **Model 4: X = Global life evaluation, M1 = Affective trust, M2 = Cognitive trust, M3 = Negative affect, M4 = Positive affect, Y = Team** | | | | | | | |
| Global life evaluation → Affective trust | Direct effects (a1) | | .067 | | .031 | .104 | |
| Global life evaluation → Cognitive trust | Direct effects (a2) | | .017 | | -.015 | .049 | |
| Global life evaluation → Negative affect | Direct effects (a3) | | .000 | | -.038 | .039 | |
| Global life evaluation → Positive affect | Direct effects (a4) | | .052 | | .015 | .089 | |
| Affective trust → Team | Direct effects (b1) | | .123 | | .061 | .185 | |
| Cognitive trust → Team | Direct effects (b2) | | .212 | | .132 | .291 | |
| Negative affect → Team | Direct effects (b3) | | -.091 | | -.147 | -.036 | |
| Positive affect → Team | Direct effects (b4) | | .203 | | .134 | .273 | |
| Global life evaluation → Team | Direct effects (c’) | | .005 | | -.021 | .031 | |
| Global life evaluation → Affective trust → Team | Indirect effects (a1×b1) | | .008 | | .002 | .016 | |
| Global life evaluation → Cognitive trust → Team | Indirect effects (a2×b2) | | .003 | | -.003 | .011 | |
| Global life evaluation → Negative affect → Team | Indirect effects (a3×b3) | | -.000 | | -.004 | .003 | |
| Global life evaluation → Positive affect → Team | Indirect effects (a4×b4) | | .010 | | .002 | .021 | |
| Global life evaluation → Team | Total effects (c) | | .027 | | -.003 | .059 | |
| Age → Team | Covariates | | -.003 | | -.008 | .000 | |
| Gender (1 = Female) → Team | Covariates | | .051 | | -.035 | .139 | |
| Job position (1 = Manager) → Team | Covariates | | -.008 | | -.099 | .084 | |
| Education → Team | Covariates | | -.015 | | -.047 | .015 | |
| Job tenure → Team | Covariates | | .002 | | -.006 | .010 | |
| **Model 5: X = Global life evaluation, M1 = Affective trust, M2 = Cognitive trust, M3 = Negative affect, M4 = Positive affect, Y = Organization** | | | | | | | |
| Global life evaluation → Affective trust | Direct effects (a1) | | .067 | | .031 | | .104 |
| Global life evaluation → Cognitive trust | Direct effects (a2) | | .017 | | -.015 | | .049 |
| Global life evaluation → Negative affect | Direct effects (a3) | | .000 | | -.038 | | .039 |
| Global life evaluation → Positive affect | Direct effects (a4) | | .052 | | .015 | | .089 |
| Affective trust → Organization | Direct effects (b1) | | .079 | | .015 | | .143 |
| Cognitive trust → Organization | Direct effects (b2) | | .276 | | .193 | | .358 |
| Negative affect → Organization | Direct effects (b3) | | -.049 | | -.105 | | .007 |
| Positive affect → Organization | Direct effects (b4) | | .209 | | .137 | | .281 |
| Global life evaluation → Organization | Direct effects (c’) | | -.012 | | -.039 | | .014 |
| Global life evaluation → Affective trust → Organization | Indirect effects (a1×b1) | | .005 | | .000^d^ | | .012 |
| Global life evaluation → Cognitive trust → Organization | Indirect effects (a2×b2) | | .004 | | -.004 | | .014 |
| Global life evaluation → Negative affect → Organization | Indirect effects (a3×b3) | | .000 | | -.002 | | .002 |
| Global life evaluation → Positive affect → Organization | Indirect effects (a4×b4) | | .011 | | .002 | | .021 |
| Global life evaluation → Organization | Total effects (c) | | .008 | | -.023 | | .040 |
| Age → Organization | Covariates | | .002 | | -.002 | | .007 |
| Gender (1 = Female) → Organization | Covariates | | .051 | | -.039 | | .141 |
| Job position (1 = Manager) → Organization | Covariates | | .031 | | -.062 | | .126 |
| Education → Organization | Covariates | | -.011 | | -.044 | | .020 |
| Job tenure → Organization | Covariates | | .002 | | -.006 | | .011 |

Notes: ^a^ The full number is .0007. ^b^ The full number is .0009. ^c^ The full number is .0006. ^d^ The full number is .0002.

**Section 9: Results of mediation analysis of the relationship between life meaning and perceptions of happy colleagues’ performance**

| **Pathway** | **Effect relationship** | **B** | **Bootstrap 95% CI** | | |
| --- | --- | --- | --- | --- | --- |
|  |  |  | **Lower** | | **Upper** |
| **Model 1: X = Life meaning, M1 = Affective trust, M2 = Cognitive trust, M3 = Negative affect, M4 = Positive affect, Y = Job** | | | | | |
| Life meaning → Affective trust | Direct effects (a1) | .083 | .057 | .110 | |
| Life meaning → Cognitive trust | Direct effects (a2) | .073 | .049 | .096 | |
| Life meaning → Negative affect | Direct effects (a3) | -.062 | -.090 | -.033 | |
| Life meaning → Positive affect | Direct effects (a4) | .095 | .069 | .122 | |
| Affective trust → Job | Direct effects (b1) | .001 | -.058 | .061 | |
| Cognitive trust → Job | Direct effects (b2) | .269 | .193 | .346 | |
| Negative affect → Job | Direct effects (b3) | -.053 | -.106 | -.000^a^ | |
| Positive affect → Job | Direct effects (b4) | .230 | .163 | .297 | |
| Life meaning → Job | Direct effects (c’) | .016 | -.003 | .035 | |
| Life meaning → Affective trust → Job | Indirect effects (a1×b1) | .000 | -.005 | .005 | |
| Life meaning → Cognitive trust → Job | Indirect effects (a2×b2) | .019 | .011 | .029 | |
| Life meaning → Negative affect → Job | Indirect effects (a3×b3) | .003 | .000^b^ | .007 | |
| Life meaning → Positive affect → Job | Indirect effects (a4×b4) | .022 | .012 | .032 | |
| Life meaning → Job | Total effects (c) | .061 | .039 | .083 | |
| Age → Job | Covariates | -.001 | -.005 | .002 | |
| Gender (1 = Female) → Job | Covariates | .067 | -.016 | .152 | |
| Job position (1 = Manager) → Job | Covariates | -.030 | -.118 | .057 | |
| Education → Job | Covariates | -.043 | -.073 | -.014 | |
| Job tenure → Job | Covariates | .005 | -.003 | .013 | |
| **Model 2: X = Life meaning , M1 = Affective trust, M2 = Cognitive trust, M3 = Negative affect, M4 = Positive affect, Y = Career** | | | | | |
| Life meaning → Affective trust | Direct effects (a1) | .083 | .057 | .110 | |
| Life meaning → Cognitive trust | Direct effects (a2) | .073 | .049 | .096 | |
| Life meaning → Negative affect | Direct effects (a3) | -.062 | -.090 | -.033 | |
| Life meaning → Positive affect | Direct effects (a4) | .095 | .069 | .122 | |
| Affective trust → Career | Direct effects (b1) | .088 | .009 | .167 | |
| Cognitive trust → Career | Direct effects (b2) | .227 | .126 | .328 | |
| Negative affect → Career | Direct effects (b3) | .007 | -.062 | .077 | |
| Positive affect → Career | Direct effects (b4) | .219 | .131 | .307 | |
| Life meaning → Career | Direct effects (c’) | .030 | .004 | .055 | |
| Life meaning → Affective trust → Career | Indirect effects (a1×b1) | .007 | .000^c^ | .015 | |
| Life meaning → Cognitive trust → Career | Indirect effects (a2×b2) | .016 | .007 | .028 | |
| Life meaning → Negative affect → Career | Indirect effects (a3×b3) | -.000 | -.005 | .004 | |
| Life meaning → Positive affect → Career | Indirect effects (a4×b4) | .021 | .010 | .032 | |
| Life meaning → Career | Total effects (c) | .074 | .047 | .101 | |
| Age → Career | Covariates | -.005 | -.011 | .000 | |
| Gender (1 = Female) → Career | Covariates | -.030 | -.141 | .080 | |
| Job position (1 = Manager) → Career | Covariates | -.002 | -.118 | .112 | |
| Education → Career | Covariates | -.033 | -.072 | .005 | |
| Job tenure → Career | Covariates | .011 | .000^d^ | .022 | |
| **Model 3: X = Life meaning, M1 = Affective trust, M2 = Cognitive trust, M3 = Negative affect, M4 = Positive affect, Y = Innovation** | | | | | |
| Life meaning → Affective trust | Direct effects (a1) | .083 | .057 | .110 | |
| Life meaning → Cognitive trust | Direct effects (a2) | .073 | .049 | .096 | |
| Life meaning → Negative affect | Direct effects (a3) | -.062 | -.090 | -.033 | |
| Life meaning → Positive affect | Direct effects (a4) | .095 | .069 | .122 | |
| Affective trust → Innovation | Direct effects (b1) | .082 | .010 | .154 | |
| Cognitive trust → Innovation | Direct effects (b2) | .292 | .200 | .385 | |
| Negative affect → Innovation | Direct effects (b3) | -.037 | -.101 | .027 | |
| Positive affect → Innovation | Direct effects (b4) | .176 | .095 | .257 | |
| Life meaning → Innovation | Direct effects (c’) | .034 | .011 | .058 | |
| Life meaning → Affective trust → Innovation | Indirect effects (a1×b1) | .006 | .000^e^ | .014 | |
| Life meaning → Cognitive trust → Innovation | Indirect effects (a2×b2) | .021 | .012 | .032 | |
| Life meaning → Negative affect → Innovation | Indirect effects (a3×b3) | .002 | -.002 | .008 | |
| Life meaning → Positive affect → Innovation | Indirect effects (a4×b4) | .016 | .007 | .028 | |
| Life meaning → Innovation | Total effects (c) | .082 | .056 | .108 | |
| Age → Innovation | Covariates | -.000 | -.005 | .004 | |
| Gender (1 = Female) → Innovation | Covariates | .065 | -.036 | .167 | |
| Job position (1 = Manager) → Innovation | Covariates | -.007 | -.113 | .098 | |
| Education → Innovation | Covariates | -.009 | -.045 | .026 | |
| Job tenure → Innovation | Covariates | .000 | -.009 | .010 | |
| **Model 4: X =Life meaning, M1 = Affective trust, M2 = Cognitive trust, M3 = Negative affect, M4 = Positive affect, Y = Team** | | | | | |
| Life meaning → Affective trust | Direct effects (a1) | .083 | .057 | .110 | |
| Life meaning → Cognitive trust | Direct effects (a2) | .073 | .049 | .096 | |
| Life meaning → Negative affect | Direct effects (a3) | -.062 | -.090 | -.033 | |
| Life meaning → Positive affect | Direct effects (a4) | .095 | .069 | .122 | |
| Affective trust → Team | Direct effects (b1) | .118 | .056 | .180 | |
| Cognitive trust → Team | Direct effects (b2) | .209 | .129 | .288 | |
| Negative affect → Team | Direct effects (b3) | -.089 | -.144 | -.033 | |
| Positive affect → Team | Direct effects (b4) | .198 | .128 | .268 | |
| Life meaning → Team | Direct effects (c’) | .020 | .000^f^ | .040 | |
| Life meaning → Affective trust → Tem | Indirect effects (a1×b1) | .009 | .003 | .017 | |
| Life meaning → Cognitive trust → Team | Indirect effects (a2×b2) | .015 | .008 | .024 | |
| Life meaning → Negative affect → Team | Indirect effects (a3×b3) | .005 | .001 | .011 | |
| Life meaning → Positive affect → Team | Indirect effects (a4×b4) | .019 | .009 | .031 | |
| Life meaning → Team | Total effects (c) | .070 | .047 | .093 | |
| Age → Team | Covariates | -.004 | -.008 | .000 | |
| Gender (1 = Female) → Team | Covariates | .048 | -.039 | .135 | |
| Job position (1 = Manager) → Team | Covariates | -.016 | -.107 | .074 | |
| Education → Team | Covariates | -.017 | -.047 | .013 | |
| Job tenure → Team | Covariates | .001 | -.006 | .010 | |
| **Model 5: X = Life meaning, M1 = Affective trust, M2 = Cognitive trust, M3 = Negative affect, M4 = POSITIVE affect, Y = Organization** | | | | | |
| Life meaning → Affective trust | Direct effects (a1) | .083 | .057 | .110 | |
| Life meaning → Cognitive trust | Direct effects (a2) | .073 | .049 | .096 | |
| Life meaning → Negative affect | Direct effects (a3) | -.062 | -.090 | -.033 | |
| Life meaning → Positive affect | Direct effects (a4) | .095 | .069 | .122 | |
| Affective trust → Organization | Direct effects (b1) | .073 | .009 | .137 | |
| Cognitive trust → Organization | Direct effects (b2) | .277 | .195 | .359 | |
| Negative affect → Organization | Direct effects (b3) | -.048 | -.105 | .008 | |
| Positive affect → Organization | Direct effects (b4) | .203 | .132 | .275 | |
| Life meaning → Organization | Direct effects (c’) | .011 | -.009 | .032 | |
| Life meaning → Affective trust → Organization | Indirect effects (a1×b1) | .006 | -.000 | .013 | |
| Life meaning → Cognitive trust → Organization | Indirect effects (a2×b2) | .020 | .012 | .030 | |
| Life meaning → Negative affect → Organization | Indirect effects (a3×b3) | .003 | -.000 | .008 | |
| Life meaning → Positive affect → Organization | Indirect effects (a4×b4) | .019 | .010 | .030 | |
| Life meaning → Organization | Total effects (c) | .060 | .036 | .084 | |
| Age → Organization | Covariates | .002 | -.002 | .006 | |
| Gender (1 = Female) → Organization | Covariates | .047 | -.042 | .137 | |
| Job position (1 = Manager) → Organization | Covariates | .017 | -.075 | .111 | |
| Education → Organization | Covariates | -.017 | -.048 | .014 | |
| Job tenure → Organization | Covariates | .001 | -.007 | .010 | |

Notes: ^a^ The full number is -.0001. ^b^ The full number is .0000. ^c^ The full number is .0003. ^d^ The full number is .0003. ^e^ The full number is .0005. ^f^ The full number is .0008.

**Section 10: Results of mediation analysis of the relationship between valuing happiness and perceptions of happy colleagues’ performance**

| **Pathway** | **Effect relationship** | **B** | **Bootstrap 95% CI** | |
| --- | --- | --- | --- | --- |
|  |  |  | **Lower** | **Upper** |
| **Model 1: X = Value happiness, M1 = Affective trust, M2 = Cognitive trust, M3 = Negative affect, M4 = Positive affect, Y = Job** | | | | |
| Value happiness → Affective trust | Direct effects (a1) | .169 | .122 | .215 |
| Value happiness → Cognitive trust | Direct effects (a2) | .134 | .092 | .176 |
| Value happiness → Negative affect | Direct effects (a3) | -.065 | -.116 | -.014 |
| Value happiness → Positive affect | Direct effects (a4) | .185 | .138 | .232 |
| Affective trust → Job | Direct effects (b1) | -.004 | -.063 | .055 |
| Cognitive trust → Job | Direct effects (b2) | .266 | .190 | .342 |
| Negative affect → Job | Direct effects (b3) | -.058 | -.111 | -.005 |
| Positive affect → Job | Direct effects (b4) | .219 | .152 | .286 |
| Value happiness → Job | Direct effects (c’) | .064 | .030 | .098 |
| Value happiness → Affective trust → Job | Indirect effects (a1×b1) | -.000 | -.011 | .009 |
| Value happiness → Cognitive trust → Job | Indirect effects (a2×b2) | .035 | .020 | .054 |
| Value happiness → Negative affect → Job | Indirect effects (a3×b3) | .003 | .000^a^ | .009 |
| Value happiness → Positive affect → Job | Indirect effects (a4×b4) | .040 | .022 | .061 |
| Value happiness → Job | Total effects (c) | .143 | .104 | .182 |
| Age → Job | Covariates | -.001 | -.005 | .003 |
| Gender (1 = Female) → Job | Covariates | .079 | -.004 | .162 |
| Job position (1 = Manager) → Job | Covariates | -.039 | -.125 | .047 |
| Education → Job | Covariates | -.038 | -.067 | -.009 |
| Job tenure → Job | Covariates | .006 | -.002 | .014 |
| **Model 2: X = Value happiness, M1 = Affective trust, m2 = Cognitive trust, M3 = Negative affect, m4 = Positive affect, Y = Career** | | | | |
| Value happiness → Affective trust | Direct effects (a1) | .169 | .122 | .215 |
| Value happiness → Cognitive trust | Direct effects (a2) | .134 | .092 | .176 |
| Value happiness → Negative affect | Direct effects (a3) | -.065 | -.116 | -.014 |
| Value happiness → Positive affect | Direct effects (a4) | .185 | .138 | .232 |
| Affective trust → Career | Direct effects (b1) | .081 | .003 | .159 |
| Cognitive trust → Career | Direct effects (b2) | .222 | .122 | .323 |
| Negative affect → Career | Direct effects (b3) | -.001 | -.071 | .068 |
| Positive affect → Career | Direct effects (b4) | .205 | .117 | .293 |
| Value happiness → Career | Direct effects (c’) | .095 | .050 | .140 |
| Value happiness → Affective trust → Career | Indirect effects (a1×b1) | .013 | -.000 | .029 |
| Value happiness → Cognitive trust → Career | Indirect effects (a2×b2) | .030 | .013 | .050 |
| Value happiness → Negative affect → Career | Indirect effects (a3×b3) | .000 | -.005 | .005 |
| Value happiness → Positive affect → Career | Indirect effects (a4×b4) | .038 | .018 | .060 |
| Value happiness → Career | Total effects (c) | .177 | .130 | .224 |
| Age → Career | Covariates | -.005 | -.010 | .000 |
| Gender (1 = Female) → Career | Covariates | -.012 | -.122 | .097 |
| Job position (1 = Manager) → Career | Covariates | -.012 | -.126 | .102 |
| Education → Career | Covariates | -.024 | -.063 | .013 |
| Job tenure → Career | Covariates | .012 | .001 | .023 |
| **Model 3: X = Value happiness, M1 = Affective trust, M2 = Cognitive trust, M3 = Negative affect, M4 = Positive affect, Y = Innovation** | | | | |
| Value happiness → Affective trust | Direct effects (a1) | .169 | .122 | .215 |
| Value happiness → Cognitive trust | Direct effects (a2) | .134 | .092 | .176 |
| Value happiness → Negative affect | Direct effects (a3) | -.065 | -.116 | -.014 |
| Value happiness → Positive affect | Direct effects (a4) | .185 | .138 | .232 |
| Affective trust → Innovation | Direct effects (b1) | .073 | .002 | .145 |
| Cognitive trust → Innovation | Direct effects (b2) | .287 | .195 | .379 |
| Negative affect → Innovation | Direct effects (b3) | -.047 | -.111 | .016 |
| Positive affect → Innovation | Direct effects (b4) | .159 | .079 | .240 |
| Value happiness → Innovation | Direct effects (c’) | .112 | .071 | .153 |
| Value happiness → Affective trust → Innovation | Indirect effects (a1×b1) | .012 | -.000 | .027 |
| Value happiness → Cognitive trust → Innovation | Indirect effects (a2×b2) | .038 | .021 | .059 |
| Value happiness → Negative affect → Innovation | Indirect effects (a3×b3) | .003 | -.001 | .010 |
| Value happiness → Positive affect → Innovation | Indirect effects (a4×b4) | .029 | .011 | .050 |
| Value happiness → Innovation | Total effects (c) | .196 | .151 | .241 |
| Age → Innovation | Covariates | -.000 | -.005 | .005 |
| Gender (1 = Female) → Innovation | Covariates | .086 | -.014 | .187 |
| Job position (1 = Manager) → Innovation | Covariates | -.019 | -.123 | .085 |
| Education → Innovation | Covariates | .000 | -.034 | .036 |
| Job tenure → Innovation | Covariates | .002 | -.008 | .011 |
| **Model 4: X =**  **Value happiness, M1 = Affective trust, M2 = Cognitive trust, M3 = Negative affect, M4 = Positive affect, Y = Team** | | | | |
| Value happiness → Affective trust | Direct effects (a1) | .169 | .122 | .215 |
| Value happiness → Cognitive trust | Direct effects (a2) | .134 | .092 | .176 |
| Value happiness → Negative affect | Direct effects (a3) | -.065 | -.116 | -.014 |
| Value happiness → Positive affect | Direct effects (a4) | .185 | .138 | .232 |
| Affective trust → Team | Direct effects (b1) | .115 | .053 | .177 |
| Cognitive trust → Team | Direct effects (b2) | .207 | .127 | .286 |
| Negative affect → Team | Direct effects (b3) | -.094 | -.149 | -.039 |
| Positive affect → Team | Direct effects (b4) | .191 | .122 | .261 |
| Value happiness → Team | Direct effects (c’) | .052 | .017 | .088 |
| Value happiness → Affective trust → Team | Indirect effects (a1×b1) | .019 | .006 | .034 |
| Value happiness → Cognitive trust → Team | Indirect effects (a2×b2) | .027 | .014 | .044 |
| Value happiness → Negative affect → Team | Indirect effects (a3×b3) | .006 | .000^b^ | .014 |
| Value happiness → Positive affect → Team | Indirect effects (a4×b4) | .035 | .017 | .057 |
| Value happiness → Team | Total effects (c) | .141 | .101 | .182 |
| Age → Team | Covariates | -.003 | -.008 | .000 |
| Gender (1 = Female) → Team | Covariates | .058 | -.028 | .146 |
| Job position (1 = Manager) → Team | Covariates | -.019 | -.109 | .071 |
| Education → Team | Covariates | -.011 | -.042 | .018 |
| Job tenure → Team | Covariates | .002 | -.006 | .011 |
| **Model 5: X = Value happiness, M1 = Affective trust, M2 = Cognitive trust, M3 = Negative affect, M4 = Positive affect, Y = Organization** | | | | |
| Value happiness → Affective trust | Direct effects (a1) | .169 | .122 | .215 |
| Value happiness → Cognitive trust | Direct effects (a2) | .134 | .092 | .176 |
| Value happiness → Negative affect | Direct effects (a3) | -.065 | -.116 | -.014 |
| Value happiness → Positive affect | Direct effects (a4) | .185 | .138 | .232 |
| Affective trust → Organization | Direct effects (b1) | .072 | .008 | .136 |
| Cognitive trust → Organization | Direct effects (b2) | .276 | .194 | .358 |
| Negative affect → Organization | Direct effects (b3) | -.050 | -.107 | .006 |
| Positive affect → Organization | Direct effects (b4) | .201 | .129 | .273 |
| Value happiness → Organization | Direct effects (c’) | .024 | -.012 | .061 |
| Value happiness → Affective trust → Organization | Indirect effects (a1×b1) | .012 | -.000 | .026 |
| Value happiness → Cognitive trust → Organization | Indirect effects (a2×b2) | .037 | .021 | .055 |
| Value happiness → Negative affect → Organization | Indirect effects (a3×b3) | .003 | -.000 | .009 |
| Value happiness → Positive affect → Organization | Indirect effects (a4×b4) | .037 | .019 | .058 |
| Value happiness → Organization | Total effects (c) | .114 | .072 | .156 |
| Age → Organization | Covariates | .002 | -.002 | .007 |
| Gender (1 = Female) → Organization | Covariates | .053 | -.036 | .143 |
| Job position (1 = Manager) → Organization | Covariates | .017 | -.076 | .111 |
| Education → Organization | Covariates | -.014 | -.045 | .017 |
| Job tenure → Organization | Covariates | .002 | -.006 | .011 |

Note: ^a^ The full number is .0000. ^b^ The full number is .0007.

**Section 11: Results of mediation analysis of the relationship between** **job satisfaction and perceptions of happy colleagues’ performance**

| **Pathway** | **Effect relationship** | **B** | **Bootstrap 95% CI** | |
| --- | --- | --- | --- | --- |
|  |  |  | **Lower** | **Upper** |
| **Model 1: X = Job satisfaction, M1 = Affective trust, M2 = Cognitive trust, M3 = Negative affect, M4 = Positive affect, Y = Job** | | | | |
| Job satisfaction → Affective trust | Direct effects (a1) | .090 | .065 | .114 |
| Job satisfaction → Cognitive trust | Direct effects (a2) | .056 | .034 | .079 |
| Job satisfaction → Negative affect | Direct effects (a3) | -.042 | -.069 | -.015 |
| Job satisfaction → Positive affect | Direct effects (a4) | .094 | .069 | .119 |
| Affective trust → Job | Direct effects (b1) | .000 | -.059 | .060 |
| Cognitive trust → Job | Direct effects (b2) | .273 | .196 | .349 |
| Negative affect → Job | Direct effects (b3) | -.054 | -.107 | -.001 |
| Positive affect → Job | Direct effects (b4) | .229 | .161 | .296 |
| Job satisfaction → Job | Direct effects (c’) | .013 | -.004 | .032 |
| Job satisfaction → Affective trust → Job | Indirect effects (a1×b1) | .000 | -.005 | .005 |
| Job satisfaction → Cognitive trust → Job | Indirect effects (a2×b2) | .015 | .008 | .024 |
| Job satisfaction → Negative affect → Job | Indirect effects (a3×b3) | .002 | .000a | .005 |
| Job satisfaction → Positive affect → Job | Indirect effects (a4×b4) | .021 | .011 | .032 |
| Job satisfaction → Job | Total effects (c) | .053 | .032 | .074 |
| Age → Job | Covariates | -.001 | -.005 | .002 |
| Gender (1 = Female) → Job | Covariates | .068 | -.015 | .152 |
| Job position (1 = Manager) → Job | Covariates | -.033 | -.121 | .054 |
| Education → Job | Covariates | -.042 | -.072 | -.013 |
| Job tenure → Job | Covariates | .005 | -.002 | .013 |
| Model 2**: X = Job satisfaction, M1 = Affective trust, M2 = Cognitive trust, M3 = Negative affect, M4 = Positive affect, Y = Career** | | | | |
| Job satisfaction → Affective trust | Direct effects (a1) | .090 | .065 | .114 |
| Job satisfaction → Cognitive trust | Direct effects (a2) | .056 | .034 | .079 |
| Job satisfaction → Negative affect | Direct effects (a3) | -.042 | -.069 | -.015 |
| Job satisfaction → Positive affect | Direct effects (a4) | .094 | .069 | .119 |
| Affective trust → Career | Direct effects (b1) | .082 | .003 | .161 |
| Cognitive trust → Career | Direct effects (b2) | .235 | .134 | .336 |
| Negative affect → Career | Direct effects (b3) | .005 | -.064 | .075 |
| Positive affect → Career | Direct effects (b4) | .212 | .123 | .301 |
| Job satisfaction → Career | Direct effects (c’) | .035 | .011 | .059 |
| Job satisfaction → Affective trust → Career | Indirect effects (a1×b1) | .007 | .000^b^ | .015 |
| Job satisfaction → Cognitive trust → Career | Indirect effects (a2×b2) | .013 | .005 | .023 |
| Job satisfaction → Negative affect → Career | Indirect effects (a3×b3) | -.000 | -.003 | .003 |
| Job satisfaction → Positive affect → Career | Indirect effects (a4×b4) | .020 | .009 | .032 |
| Job satisfaction → Career | Total effects (c) | .075 | .050 | .101 |
| Age → Career | Covariates | -.005 | -.011 | .000 |
| Gender (1 = Female) → Career | Covariates | -.030 | -.141 | .080 |
| Job position (1 = Manager) → Career | Covariates | -.016 | -.132 | .100 |
| Education → Career | Covariates | -.033 | -.072 | .005 |
| Job tenure → Career | Covariates | .011 | .000 | .022 |
| Model 3: **X = Job satisfaction, M1 = Affective trust, M2 = Cognitive trust, M3 = Negative affect, M4 = Positive affect, Y =Innovation** | | | | |
| Job satisfaction → Affective trust | Direct effects (a1) | .090 | .065 | .114 |
| Job satisfaction → Cognitive trust | Direct effects (a2) | .056 | .034 | .079 |
| Job satisfaction → Negative affect | Direct effects (a3) | -.042 | -.069 | -.015 |
| Job satisfaction → Positive affect | Direct effects (a4) | .094 | .069 | .119 |
| Affective trust → Innovation | Direct effects (b1) | .079 | .007 | .152 |
| Cognitive trust → Innovation | Direct effects (b2) | .300 | .207 | .393 |
| Negative affect → Innovation | Direct effects (b3) | -.039 | -.104 | .024 |
| Positive affect → Innovation | Direct effects (b4) | .173 | .092 | .255 |
| Job satisfaction → Innovation | Direct effects (c’) | .029 | .007 | .051 |
| Job satisfaction → Affective trust → Innovation | Indirect effects (a1×b1) | .007 | .000^c^ | .014 |
| Job satisfaction → Cognitive trust → Innovation | Indirect effects (a2×b2) | .017 | .008 | .027 |
| Job satisfaction → Negative affect → Innovation | Indirect effects (a3×b3) | .001 | -.001 | .005 |
| Job satisfaction → Positive affect → Innovation | Indirect effects (a4×b4) | .016 | .006 | .028 |
| Job satisfaction → Innovation | Total effects (c) | .072 | .047 | .096 |
| Age → Innovation | Covariates | -.000 | -.005 | .004 |
| Gender (1 = Female) → Innovation | Covariates | .067 | -.034 | .168 |
| Job position (1 = Manager) → Innovation | Covariates | -.013 | -.120 | .093 |
| Education → Innovation | Covariates | -.007 | -.043 | .027 |
| Job tenure → Innovation | Covariates | .000 | -.009 | .010 |
| **Model 4: X = Job satisfaction, M1 = Affective trust, M2 = Cognitive trust, M3 = Negative affect, M4 = Positive affect, Y = Team** | | | | |
| Job satisfaction → Affective trust | Direct effects (a1) | .090 | .065 | .114 |
| Job satisfaction → Cognitive trust | Direct effects (a2) | .056 | .034 | .079 |
| Job satisfaction → Negative affect | Direct effects (a3) | -.042 | -.069 | -.015 |
| Job satisfaction → Positive affect | Direct effects (a4) | .094 | .069 | .119 |
| Affective trust → Team | Direct effects (b1) | .115 | .053 | .178 |
| Cognitive trust → Team | Direct effects (b2) | .214 | .134 | .293 |
| Negative affect → Team | Direct effects (b3) | -.090 | -.145 | -.035 |
| Positive affect → Team | Direct effects (b4) | .195 | .125 | .265 |
| Job satisfaction → Team | Direct effects (c’) | .020 | .001 | .039 |
| Job satisfaction → Affective trust → Team | Indirect effects (a1×b1) | .010 | .003 | .018 |
| Job satisfaction → Cognitive trust → Team | Indirect effects (a2×b2) | .012 | .005 | .020 |
| Job satisfaction → Negative affect → Team | Indirect effects (a3×b3) | .003 | .000^d^ | .008 |
| Job satisfaction → Positive affect → Team | Indirect effects (a4×b4) | .018 | .008 | .030 |
| Job satisfaction → Team | Total effects (c) | .065 | .043 | .087 |
| Age → Team | Covariates | -.004 | -.008 | .000 |
| Gender (1 = Female) → Team | Covariates | .048 | -.038 | .136 |
| Job position (1 = Manager) → Team | Covariates | -.022 | -.114 | .069 |
| Education → Team | Covariates | -.016 | -.047 | .013 |
| Job tenure → Team | Covariates | .001 | -.006 | .010 |
| **Model 5: X = Job satisfaction, M1 = Affective trust, M2 = Cognitive trust, M3 = Negative affect, M4 = Positive affect, Y = Organization** | | | | |
| Job satisfaction → Affective trust | Direct effects (a1) | .090 | .065 | .114 |
| Job satisfaction → Cognitive trust | Direct effects (a2) | .056 | .034 | .079 |
| Job satisfaction → Negative affect | Direct effects (a3) | -.042 | -.069 | -.015 |
| Job satisfaction → Positive affect | Direct effects (a4) | .094 | .069 | .119 |
| Affective trust → Organization | Direct effects (b1) | .073 | .008 | .137 |
| Cognitive trust → Organization | Direct effects (b2) | .279 | .197 | .361 |
| Negative affect → Organization | Direct effects (b3) | -.049 | -.106 | .007 |
| Positive affect → Organization | Direct effects (b4) | .203 | .131 | .275 |
| Job satisfaction → Organization | Direct effects (c’) | .007 | -.011 | .027 |
| Job satisfaction → Affective trust → Organization | Indirect effects (a1×b1) | .006 | -.000 | .014 |
| Job satisfaction → Cognitive trust → Organization | Indirect effects (a2×b2) | .015 | .008 | .025 |
| Job satisfaction → Negative affect → Organization | Indirect effects (a3×b3) | .002 | -.000 | .006 |
| Job satisfaction → Positive affect → Organization | Indirect effects (a4×b4) | .019 | .009 | .030 |
| Job satisfaction → Organization | Total effects (c) | .051 | .029 | .074 |
| Age → Organization | Covariates | .002 | -.002 | .006 |
| Gender (1 = Female) → Organization | Covariates | .048 | -.041 | .138 |
| Job position (1 = Manager) → Organization | Covariates | .017 | -.077 | .112 |
| Education → Organization | Covariates | -.016 | -.047 | .015 |
| Job tenure → Organization | Covariates | .002 | -.006 | .010 |

Note: ^a^ The full number is .0000. ^b^ The full number is .0000. ^c^ The full number is .0003. ^d^ The full number is .0006.

**Section 12: Results of mediation analysis of the relationship between** **→ Job meaning and perceptions of happy colleagues’ performance**

| **Pathway** | **Effect relationship** | **B** | **Bootstrap 95% CI** | |
| --- | --- | --- | --- | --- |
|  |  |  | **Lower** | **Upper** |
| **Model 1: X = Job meaning, M1 = Affective trust, M2 = Cognitive trust, M3 = Negative affect, M4 = Positive affect, Y = Job** | | | | |
| Job meaning → Affective trust | Direct effects (a1) | .070 | .049 | .091 |
| Job meaning → Cognitive trust | Direct effects (a2) | .051 | .032 | .070 |
| Job meaning → Negative affect | Direct effects (a3) | -.038 | -.061 | -.015 |
| Job meaning → Positive affect | Direct effects (a4) | .070 | .049 | .092 |
| Affective trust → Job | Direct effects (b1) | -.002 | -.062 | .057 |
| Cognitive trust → Job | Direct effects (b2) | .270 | .194 | .347 |
| Negative affect → Job | Direct effects (b3) | -.053 | -.106 | -.000 |
| Positive affect → Job | Direct effects (b4) | .228 | .161 | .295 |
| Job meaning → Job | Direct effects (c’) | .019 | .004 | .034 |
| Job meaning Affective trust → Job | Indirect effects (a1×b1) | -.000^a^ | -.004 | .004 |
| Job meaning → Cognitive trust → Job | Indirect effects (a2×b2) | .013 | .007 | .021 |
| Job meaning → Negative affect → Job | Indirect effects (a3×b3) | .002 | .000^b^ | .005 |
| Job meaning → Positive affect → Job | Indirect effects (a4×b4) | .016 | .008 | .024 |
| Job meaning → Job | Total effects (c) | .051 | .033 | .069 |
| Age → Job | Covariates | -.001 | -.005 | .002 |
| Gender (1 = Female) → Job | Covariates | .067 | -.016 | .151 |
| Job position (1 = Manager) → Job | Covariates | -.040 | -.128 | .047 |
| Education → Job | Covariates | -.046 | -.076 | -.017 |
| Job tenure → Job | Covariates | .004 | -.003 | .013 |
| **Model 2: X = Job meaning, M1 = Affective trust, M2 = Cognitive trust, M3 = Negative affect, M4 = Positive affect, Y = Career** | | | | |
| Job meaning → Affective trust | Direct effects (a1) | .070 | .049 | .091 |
| Job meaning → Cognitive trust | Direct effects (a2) | .051 | .032 | .070 |
| Job meaning → Negative affect | Direct effects (a3) | -.038 | -.061 | -.015 |
| Job meaning → Positive affect | Direct effects (a4) | .070 | .049 | .092 |
| Affective trust → Career | Direct effects (b1) | .079 | .000^c^ | .158 |
| Cognitive trust → Career | Direct effects (b2) | .229 | .129 | .330 |
| Negative affect → Career | Direct effects (b3) | .007 | -.062 | .077 |
| Positive affect → Career | Direct effects (b4) | .215 | .127 | .303 |
| Job meaning → Career | Direct effects (c’) | .038 | .018 | .058 |
| Job meaning → Affective trust → Career | Indirect effects (a1×b1) | .005 | -.000 | .012 |
| Job meaning → Cognitive trust → Career | Indirect effects (a2×b2) | .011 | .005 | .019 |
| Job meaning → Negative affect → Career | Indirect effects (a3×b3) | -.000 | -.003 | .002 |
| Job meaning → Positive affect → Career | Indirect effects (a4×b4) | .015 | .007 | .024 |
| Job meaning → Career | Total effects © | .070 | .049 | .092 |
| Age → Career | Covariates | -.005 | -.011 | .000 |
| Gender (1 = Female) → Career | Covariates | -.031 | -.141 | .078 |
| Job position (1 = Manager) → Career | Covariates | -.023 | -.139 | .091 |
| Education → Career | Covariates | -.040 | -.079 | -.001 |
| Job tenure → Career | Covariates | .010 | -.000 | .021 |
| **Model 3: X = Job meaning, M1 = Affective trust, M2 = Cognitive trust, M3 = Negative affect, M4 = Positive affect, Y =Innovation** | | | | |
| Job meaning → Affective trust | Direct effects (a1) | .070 | .049 | .091 |
| Job meaning → Cognitive trust | Direct effects (a2) | .051 | .032 | .070 |
| Job meaning → Negative affect | Direct effects (a3) | -.038 | -.061 | -.015 |
| Job meaning → Positive affect | Direct effects (a4) | .070 | .049 | .092 |
| Affective trust → Innovation | Direct effects (b1) | .076 | .004 | .149 |
| Cognitive trust → Innovation | Direct effects (b2) | .295 | .203 | .388 |
| Negative affect → Innovation | Direct effects (b3) | -.037 | -.102 | .026 |
| Positive affect → Innovation | Direct effects (b4) | .175 | .094 | .256 |
| Job meaning → Innovation | Direct effects (c’) | .033 | .015 | .052 |
| Job meaning → Affective trust → Innovation | Indirect effects (a1×b1) | .005 | .000^d^ | .011 |
| Job meaning → Cognitive trust → Innovation | Indirect effects (a2×b2) | .015 | .008 | .023 |
| Job meaning → Negative affect → Innovation | Indirect effects (a3×b3) | .001 | -.001 | .005 |
| Job meaning → Positive affect → Innovation | Indirect effects (a4×b4) | .012 | .005 | .020 |
| Job meaning → Innovation | Total effects © | .068 | .047 | .088 |
| Age → Innovation | Covariates | -.000 | -.005 | .004 |
| Gender (1 = Female) → Innovation | Covariates | .066 | -.035 | .167 |
| Job position (1 = Manager) → Innovation | Covariates | -.021 | -.127 | .085 |
| Education → Innovation | Covariates | -.014 | -.049 | .021 |
| Job tenure → Innovation | Covariates | .000 | -.009 | .010 |
| **Model 4: X = Job meaning, M1 = Affective trust, M2 = Cognitive trust, M3 = Negative affect, M4 = Positive affect, Y = Team** | | | | |
| Job meaning → Affective trust | Direct effects (a1) | .070 | .049 | .091 |
| Job meaning → Cognitive trust | Direct effects (a2) | .051 | .032 | .070 |
| Job meaning → Negative affect | Direct effects (a3) | -.038 | -.061 | -.015 |
| Job meaning → Positive affect | Direct effects (a4) | .070 | .049 | .092 |
| Affective trust → Team | Direct effects (b1) | .114 | .052 | .176 |
| Cognitive trust → Team | Direct effects (b2) | .210 | .131 | .290 |
| Negative affect → Team | Direct effects (b3) | -.089 | -.144 | -.034 |
| Positive affect → Team | Direct effects (b4) | .197 | .127 | .266 |
| Job meaning → Team | Direct effects (c’) | .021 | .005 | .037 |
| Job meaning → Affective trust → Team | Indirect effects (a1×b1) | .008 | .002 | .014 |
| Job meaning → Cognitive trust → Team | Indirect effects (a2×b2) | .010 | .005 | .017 |
| Job meaning → Negative affect → Team | Indirect effects (a3×b3) | .003 | .000^e^ | .007 |
| Job meaning → Positive affect → Team | Indirect effects (a4×b4) | .013 | .006 | .022 |
| Job meaning → Team | Total effects © | .058 | .039 | .076 |
| Age → Team | Covariates | -.003 | -.008 | .000 |
| Gender (1 = Female) → Team | Covariates | .048 | -.038 | .135 |
| Job position (1 = Manager) → Team | Covariates | -.026 | -.117 | .065 |
| Education → Team | Covariates | -.020 | -.051 | .010 |
| Job tenure → Team | Covariates | .001 | -.007 | .010 |
| **Model 5: X = Job meaning, M1 = Affective trust, M2 = Cognitive trust, M3 = Negative affect, M4 = Positive affect, Y = Organization** | | | | |
| Job meaning → Affective trust | Direct effects (a1) | .070 | .049 | .091 |
| Job meaning → Cognitive trust | Direct effects (a2) | .051 | .032 | .070 |
| Job meaning → Negative affect | Direct effects (a3) | -.038 | -.061 | -.015 |
| Job meaning → Positive affect | Direct effects (a4) | .070 | .049 | .092 |
| Affective trust → Organization | Direct effects (b1) | .070 | .006 | .134 |
| Cognitive trust → Organization | Direct effects (b2) | .278 | .196 | .359 |
| Negative affect → Organization | Direct effects (b3) | -.048 | -.105 | .008 |
| Positive affect → Organization | Direct effects (b4) | .202 | .131 | .274 |
| Job meaning → Organization | Direct effects (c’) | .013 | -.003 | .029 |
| Job meaning → Affective trust → Organization | Indirect effects (a1×b1) | .005 | -.000 | .010 |
| Job meaning → Cognitive trust → Organization | Indirect effects (a2×b2) | .014 | .008 | .022 |
| Job meaning → Negative affect → Organization | Indirect effects (a3×b3) | .001 | -.000 | .005 |
| Job meaning → Positive affect → Organization | Indirect effects (a4×b4) | .014 | .007 | .022 |
| Job meaning → Organization | Total effects (c) | .048 | .029 | .067 |
| Age → Organization | Covariates | .002 | -.002 | .006 |
| Gender (1 = Female) → Organization | Covariates | .047 | -.042 | .137 |
| Job position (1 = Manager) → Organization | Covariates | .011 | -.083 | .105 |
| Education → Organization | Covariates | -.019 | -.051 | .012 |
| Job tenure → Organization | Covariates | .001 | -.007 | .010 |

Note: ^a^ The full number is -.0002.  ^b^ The full number is .0000. ^c^ The full number is .0007. ^d^ The full number is .0000. ^e^ The full number is .0007.
